# Navigating the 16-dimensional Hilbert space of a high-spin donor qudit with electric and magnetic fields

**DOI:** 10.1038/s41467-024-45368-y

**Published:** 2024-02-14

**Authors:** Irene Fernández de Fuentes, Tim Botzem, Mark A. I. Johnson, Arjen Vaartjes, Serwan Asaad, Vincent Mourik, Fay E. Hudson, Kohei M. Itoh, Brett C. Johnson, Alexander M. Jakob, Jeffrey C. McCallum, David N. Jamieson, Andrew S. Dzurak, Andrea Morello

**Affiliations:** 1 School of Electrical Engineering and Telecommunication, UNSW Sydney, Sydney, NSW Australia; 2 Diraq, Sydney, NSW Australia; 3 School of Fundamental Science and Technology, Keio University, Yokohama, Japan; 4 School of Science, RMIT University, Melbourne, VIC Australia; 5 School of Physics, University of Melbourne, Melbourne, VIC Australia

**Keywords:** Quantum information, Qubits

## Abstract

Efficient scaling and flexible control are key aspects of useful quantum computing hardware. Spins in semiconductors combine quantum information processing with electrons, holes or nuclei, control with electric or magnetic fields, and scalable coupling via exchange or dipole interaction. However, accessing large Hilbert space dimensions has remained challenging, due to the short-distance nature of the interactions. Here, we present an atom-based semiconductor platform where a 16-dimensional Hilbert space is built by the combined electron-nuclear states of a single antimony donor in silicon. We demonstrate the ability to navigate this large Hilbert space using both electric and magnetic fields, with gate fidelity exceeding 99.8% on the nuclear spin, and unveil fine details of the system Hamiltonian and its susceptibility to control and noise fields. These results establish high-spin donors as a rich platform for practical quantum information and to explore quantum foundations.

## Abstract (web summary)

Qudits, higher-dimensional analogues of qubits, expand quantum state space for information processing using fewer physical units. Here the authors demonstrate control over a 16-dimensional Hilbert space, equivalent to four qubits, using combined electron-nuclear states of a single Sb donor atom in Si.

## Introduction

For computing purposes, one of the key properties of quantum systems is that the dimension *D* of the computational space—in this case, the Hilbert space—can grow exponentially with the number *N* of physical qubits, i.e., as *D* = 2^*N*^. Unlike in a classical computer, where each additional bit simply adds one dimension to the data array, in a quantum computer each qubit multiplies the Hilbert space dimension by two. In practice, whether this is actually the case depends upon creating maximally entangled states with high fidelity, which in turn is a delicate function of the physical layout of the qubits and the details of the interaction between them.

An alternative quantum computing paradigm starts with physical components whose intrinsic Hilbert space dimension is *d* > 2, thus called qu*d*its^1^. Using qudits, a *D*-dimensional Hilbert space can be constructed with a factor ${\log }_{2}d$ smaller number of physical units compared to the qubit case. Circuit complexity can be reduced even further; using two-qudit gates, an *N*-dimensional unitary operator *U* can be simulated using a factor ${({\log }_{2}d)}^{2}$ less gates as compared to its qubit-based counterpart^2^. General schemes exist to perform fault-tolerant operations in a way that takes advantage of a larger *d* ^3^, and to compile various quantum algorithms in a resource-efficient way^4,5^. Experimental qudit platforms can be found in optics^6,7^, superconductors^8–10^, trapped ions^11^, atomic ensembles^12^ and molecular magnets^13^.

Here we present a physical platform for high-dimensional qudit encoding in a silicon nanoelectronic device. Silicon quantum devices^14^ host spin qubits that combine exceptionally long coherence times^15^, exceeding 30 seconds in nuclear spins^16^, one- and two-qubit gate fidelities above 99%^17–20^, and compatibility with the manufacturing processes that underpin the established semiconductor industry^21^. Electron spin qubits can be controlled using both magnetic^15,22^ (electron spin resonance, ESR) and electric^18–20^ (electric dipole spin resonance, EDSR) fields; nuclear qubits are normally driven by nuclear magnetic resonance^23^ (NMR), but quadrupolar nuclei can exhibit Electric^24^ (NER) or even Acoustic^25^ (NAR) resonances. Magnetic drive lends itself to global control methods, where a spatially extended oscillating magnetic field drives multiple qubits^26,27^, whereas electric drive is easier to localise at the nanometre scale.

Our chosen qudit platform is the antimony donor in silicon, Si:^123^Sb. Our initial interest in this system was in the context of fundamental studies on quantum chaos^28^. The serendipitous discovery of nuclear electric resonance^24^ and the steady development of ideas to use high-spin nuclei in quantum information processing^29–31^ highlighted the unique opportunity to use ^123^Sb as a qudit that exploits all the benefits and flexibility of silicon quantum electronic devices.

In this work we show magnetic and electric control over the 16-dimensional Hilbert space of the combined electron and nuclear spin of the ^123^Sb donor, benchmark quantum gate fidelities, and provide a detailed understanding of the microscopic physics that governs the behaviour of this novel qudit system.

## Results

### The antimony donor

Like phosphorus^22,23^, arsenic^32^ and bismuth^33^, antimony is a group-V donor in silicon. It behaves as a hydrogenic impurity where the Coulomb potential of the nuclear charge loosely binds an electron in a 1*s*-like orbital^14^. The ^123^Sb isotope of antimony possesses a nuclear spin *I* = 7/2, with a gyromagnetic ratio *γ*_n_ = 5.55 MHz/T. The non-spherical charge distribution in the nucleus creates an electric quadrupole moment *q*_n_ = [ − 0.49, − 0.69] × 10^−28^ m^2^ ^28^. The *S* = 1/2 spin of the donor-bound electron has a gyromagnetic ratio *γ*_e_ ≈ 27.97 GHz/T, and is magnetically coupled to the nuclear spin via the Fermi contact hyperfine interaction $A\hat{{{{{{{{\boldsymbol{S}}}}}}}}}\cdot \hat{{{{{{{{\boldsymbol{I}}}}}}}}}$, with *A* = 101.52 MHz in bulk silicon.

**Fig. 1:**
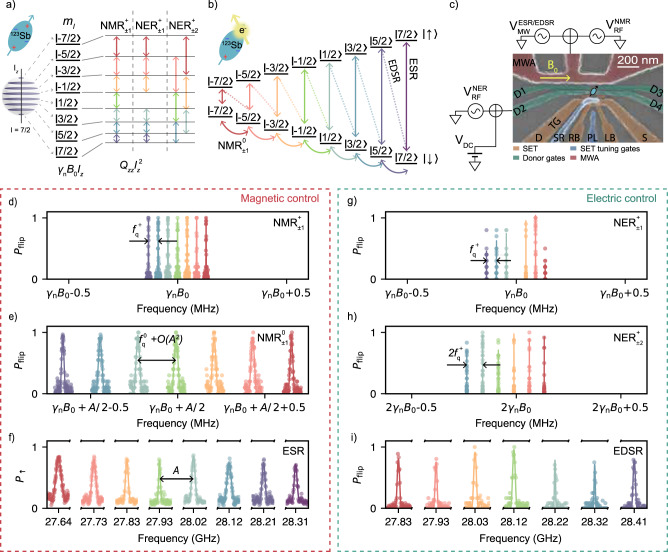
Spectrum of the ^123^Sb atom. **a** Energy diagram of the ionised ^123^Sb atom. The arrows indicate the allowed spin transitions for the different driving mechanisms, including ${\rm{NMR}}_{\pm 1}^{+}$, ${\rm{NER}}_{\pm 1}^{+}$ for Δ*m*_*I*_ = ± 1 and ${\rm{NER}}_{\pm 2}^{+}$ for Δ*m*_*I*_ = ± 2, where + denotes the charge state of the nucleus. The colours of all lines and symbols reflect the initial $\left\vert {m}_{I}-1\right\rangle$ state of each spin transition, and are used consistently across this manuscript. The Zeeman energy ${\gamma }_{{{{{{{{\rm{n}}}}}}}}}{B}_{0}{\hat{I}}_{z}$ ( ≈ 5.5 MHz in this work) yields equispaced nuclear levels, but the quadrupole coupling, written for simplicity as ${Q}_{zz}{\hat{I}}_{z}^{2}$, shifts the resonance frequencies according to *m*_*I*_ and allows their individual addressing. **b** Energy diagram of the ^123^Sb atom in the neutral charge state. The ${\rm{NMR}}_{\pm 1}^{0}$ transitions are represented by curved arrows, while the ESR is depicted by vertical solid arrows, and the EDSR is indicated by dashed arrows. **c** False-coloured scanning electron micrograph of a device identical to the one used for the experiments. The ESR, EDSR and NMR driving signals are applied to the microwave antenna (MWA), whereas the NER drives are applied to one of the open-circuited gates. The green ellipse depicts the approximate location of the implanted donor with respect to the surface gates. **d** Experimental NMR^+^ spectrum for the ionised donor, showing 7 resonant peaks. The distance between adjacent peaks is given to first order by the quadrupolar splitting ${f}_{{{{{{{{\rm{q}}}}}}}}}^{{{{{{{{\rm{+}}}}}}}}}=-44.1(2)$ kHz. **e** NMR spectrum for the neutral atom in the electron spin-down configuration, split by the quadrupolar interaction ${f}_{{{{{{{{\rm{q}}}}}}}}}^{{{{{{{{\rm{n0}}}}}}}}}=-52.5(5)$ kHz and second-order contributions of the hyperfine interaction ∝ *A*^2^/*γ*_n_*B*_0_. We use the same frequency range in the *x-*axis for panels **d**, **e** to highlight the effect of the hyperfine interaction on the separation of the resonances in the neutral case. **f** ESR spectrum, showing 8 resonance peaks depending on the nuclear projection *m*_*I*_, split to first order by the hyperfine interaction *A*. **g** ${\rm{NER}}_{\pm 1}^{+}$ spectrum for the ionised donor. The transition *m*_−1/2_ ↔ *m*_1/2_ is forbidden by NER. **h** ${\rm{NER}}_{\pm 2}^{+}$ spectrum, with frequencies ${f}_{{m}_{I}-2\leftrightarrow {m}_{I}}^{{{{{{{{{\rm{NER}}}}}}}}}^{+}}={f}_{{m}_{I}-2\to {m}_{I}-1}^{{{{{{{{{\rm{NER}}}}}}}}}^{+}}+{f}_{{m}_{I}-1\to {m}_{I}}^{{{{{{{{{\rm{NER}}}}}}}}}^{+}}$. **i** EDSR spectrum, showing 7 electron-nuclear resonances that conserve *m*_*I*_ + *m*_*S*_. In all panels from **d** to **f**, the resonance lines are power-broadened.

The charge state of the donor can be easily modified by placing it in a nanoelectronic device, where metallic electrodes lift the donor electrochemical potential *μ*_D_ above the Fermi level of a nearby charge reservoir, thus energetically favouring the weakly bound electron to leave the donor. The resulting ionised (positively charged) *D*^+^ donor, placed in a magnetic field *B*_0_ oriented along the Cartesian *z*-axis, has the following static Hamiltonian:

1 $${\hat{{{{{{{{\mathcal{H}}}}}}}}}}_{{D}^{+}}=-{B}_{0}{\gamma }_{{{{{{{{\rm{n}}}}}}}}}{\hat{I}}_{z}+\mathop{\sum}\limits_{\alpha,\beta \in \{x,y,z\}}{Q}_{{{{{{{{\rm{\alpha \beta }}}}}}}}}{\hat{I}}_{{{{{{{{\rm{\alpha }}}}}}}}}{\hat{I}}_{{{{{{{{\rm{\beta }}}}}}}}},$$

where *α*, *β* = {*x*, *y*, *z*} are Cartesian axes, ${\hat{I}}_{\alpha }$ are the corresponding 8-dimensional nuclear spin projection operators, and ${Q}_{{{{{{{{\rm{\alpha \beta }}}}}}}}}=\frac{{{{{{{{\rm{e}}}}}}}}{q}_{{{{{{{{\rm{n}}}}}}}}}{{{{{{{{\mathcal{V}}}}}}}}}_{\alpha \beta }}{2I(2I-1)h}$ is the nuclear quadrupole interaction energy, governed by the electric-field gradient (EFG) tensor ${{{{{{{{\mathcal{V}}}}}}}}}_{\alpha \beta }={\partial }^{2}V(x,y,z)/\partial \alpha \partial \beta$. The quadrupole interaction introduces an additional orientation-dependent energy shift to the nuclear Zeeman levels (Fig. 1a), allowing for the individual addressability of nuclear states even in the ionised case^32,34^. The quadrupole interaction term is determined chiefly by the lattice strain^24,32,35^, which in our device is caused by the differential thermal expansion of the aluminium gates and the silicon substrate upon cooling the device to cryogenic temperatures. Future experiments may include the ability to locally tune the strain using a piezoelectric actuator^25^.

In the charge-neutral state *D*^0^, the system Hamiltonian ${{{{{{{{\mathcal{H}}}}}}}}}_{{D}^{0}}$ becomes a 16-dimensional matrix expressed in terms of the tensor products of the electron and nuclear spin operators:

2 $${\hat{{{{{{{{\mathcal{H}}}}}}}}}}_{{D}^{0}}={B}_{0}\left(-{\gamma }_{{{{{{{{\rm{n}}}}}}}}}{\hat{I}}_{z}+{\gamma }_{{{{{{{{\rm{e}}}}}}}}}{\hat{S}}_{z}\right)+A\hat{{{{{{{{\boldsymbol{S}}}}}}}}}\cdot \hat{{{{{{{{\boldsymbol{I}}}}}}}}}+\mathop{\sum}\limits_{\alpha \beta \in \{x,y,z\}}{Q}_{{{{{{{{\rm{\alpha \beta }}}}}}}}}{\hat{I}}_{{{{{{{{\rm{\alpha }}}}}}}}}{\hat{I}}_{{{{{{{{\rm{\beta }}}}}}}}}.$$

We operate the device in a magnetic field *B*_0_ ≈ 1 T, which ensures that the eigenstates of ${\hat{{{{{{{{\mathcal{H}}}}}}}}}}_{{D}^{+}}$ (Fig. 1a) are well approximated by the eigenstates $\left\vert {m}_{I}\right\rangle$ of ${\hat{I}}_{z}$ (*m*_*I*_ = − 7/2, − 5/2. . . , + 7/2) because *γ*_n_*B*_0_ ≫ *Q*_*αβ*_, and the eigenstates of ${\hat{{{{{{{{\mathcal{H}}}}}}}}}}_{{D}^{0}}$ (Fig. 1b) are approximately the tensor products of $\left\vert {m}_{I}\right\rangle$ with the eigenstates $\{\left\vert \downarrow \right\rangle,\left\vert \uparrow \right\rangle \}$ of ${\hat{S}}_{z}$ because *γ*_e_*B*_0_ ≫ *A* ≫ *Q*_*αβ*_. The latter condition implies ${\hat{{{{{{{{\mathcal{H}}}}}}}}}}_{{D}^{0}}\approx {B}_{0}(-{\gamma }_{{{{{{{{\rm{n}}}}}}}}}{\hat{I}}_{z}+{\gamma }_{{{{{{{{\rm{e}}}}}}}}}{\hat{S}}_{z})+A{\hat{S}}_{z}{\hat{I}}_{z}$ ensuring that the nuclear spin operator approximately commutes with the electron-nuclear interaction. This condition allows for nearly quantum nondemolition (QND) readout of the nuclear spin via the electron spin ancilla^23^ (see Supplementary Materials, Section 1 for deviations from the QND condition). The benefit of nuclear QND readout is the key reason why we chose ^123^Sb instead of ^209^Bi as our preferred high-spin donor. ^209^Bi has an even higher nuclear spin, *I*_Bi_ = 9/2, but also an order of magnitude larger hyperfine coupling, *A*_Bi_ = 1.475 GHz. This creates interesting spin physics phenomena related to the strong electron-nuclear mixing^33,36^. However, in single-donor experiments where the nuclear spin is read out via the ancilla electron, the stronger hyperfine coupling introduces a measurement back-action (in other words, a deviation from the QND measurement condition) of order ${(A/{\gamma }_{e}{B}_{0})}^{2}$ ^37^, which is thus over two orders of magnitude larger in ^209^Bi compared to ^123^Sb.

A key feature of this work is that coherent transitions between the ^123^Sb spin eigenstates can be induced by both magnetic and electric fields, on both the electron and the nuclear spin. Electron spin resonance (ESR)^22^ is achieved by adding the driving term ${\hat{{{{{{{{\mathcal{H}}}}}}}}}}^{{{{{{{{\rm{ESR}}}}}}}}}={B}_{1}{\gamma }_{{{{{{{{\rm{e}}}}}}}}}{\hat{S}}_{x}\cos (2\pi {f}_{{m}_{I}}^{{{{{{{{\rm{ESR}}}}}}}}}t)$ to ${\hat{{{{{{{{\mathcal{H}}}}}}}}}}_{{D}^{0}}$, where *B*_1_ is the amplitude of an oscillating magnetic field at one of the eight resonance frequencies ${f}_{{m}_{I}}^{{{{{{{{\rm{ESR}}}}}}}}}$ determined by the nuclear spin projection *m*_*I*_. Similarly, nuclear magnetic resonance (NMR)^23^ requires a magnetic drive term ${\hat{{{{{{{{\mathcal{H}}}}}}}}}}^{{{{{{{{\rm{NMR}}}}}}}}}={B}_{1}{\gamma }_{{{{{{{{\rm{n}}}}}}}}}{\hat{I}}_{x}\cos (2\pi {f}_{{m}_{I}-1\leftrightarrow {m}_{I}}^{{{{{{{{\rm{NMR}}}}}}}}}t)$, applicable to both the neutral (${\rm{NMR}}_{\pm 1}^{0}$) and the ionised (${\rm{NMR}}_{\pm 1}^{+}$) case. The ± 1 subscript indicates that such transitions change the nuclear spin projection by one quantum of angular momentum, i.e., Δ*m*_*I*_ = ± 1.

Electrically driven spin transitions can be obtained in two ways. One, involving the combined state of electron and nucleus, is the high-spin generalisation of the ‘flip-flop’ transition demonstrated recently in the *I* = 1/2 ^31^P system^38^. An oscillating electric field ${E}_{1}\cos (2\pi {f}_{{m}_{I}-1\leftrightarrow {m}_{I}}^{{{{{{{{\rm{EDSR}}}}}}}}}t)$ induces electric dipole spin resonance transitions (EDSR) in the neutral donor by time-dependently modulating the hyperfine interaction $A({E}_{1}){\hat{S}}_{\pm }{\hat{I}}_{\mp }$ via the Stark effect^39^, where the ± subindices indicate the rising and lowering operators, respectively. This mechanism preserves the total angular momentum of the combined electron-nuclear states. Therefore, the EDSR transitions appear as diagonal (dashed) lines in Fig. 1b. The second electrical transition, called nuclear electric resonance (NER)^24^ acts on the nucleus alone. It exploits the modulation of electric quadrupole coupling terms involving the operators ${\hat{I}}_{z}{\hat{I}}_{\pm }$ for transitions with Δ*m*_*I*_ = ± 1 (NER_±1_), and ${\hat{I}}_{\pm }^{2}$ for transitions with Δ*m*_*I*_ = ± 2 (NER_±2_). The microscopic mechanism by which the electric field ${E}_{1}\cos (2\pi {f}_{{m}_{I}-1\leftrightarrow {m}_{I}}^{{{{{{{{\rm{NER}}}}}}}}}t)$ creates a time-dependent electric-field gradient at the nucleus was understood to arise from the distortion of the atomic bond orbitals, in a lattice site lacking point inversion symmetry^24^. The energy level structure of the neutral and ionised ^123^Sb results in a total of 54 resonant transitions, the frequencies of which are listed in Table 1.

**Table 1:** Resonance frequencies, nuclear-state dependent scaling coefficients and Rabi rates for the different spin driving mechanisms of the ^123^Sb donor, including electric (NER_±1,±2_, EDSR) and magnetic (NMR, ESR) control

Resonance frequencies	Nuclear-state dependent driving coefficients	Rabi rates
${f}_{{m}_{I}-1\leftrightarrow {m}_{I}}^{{{{{{\rm{NM}}}}}}{{{{{{\rm{R}}}}}}}^{+}}={\gamma }_{{{{{{\rm{n}}}}}}}{B}_{0}+\left({m}_{I}-\frac{1}{2}\right){f}_{{{{{{\rm{q}}}}}}}^{+}$	${\zeta }_{{m}_{I}-1\leftrightarrow {m}_{I}}^{{{{{{\rm{NMR}}}}}}}=\frac{1}{2}\sqrt{I\left(I+1\right)-{m}_{I}({m}_{I}-1)}$	${f}_{{m}_{I}-1\leftrightarrow {m}_{I}}^{{{{{{\rm{Rabi}}}}}},{{{{{\rm{NM}}}}}}{{{{{{\rm{R}}}}}}}^{+}}={\gamma }_{{{{{{\rm{n}}}}}}}{B}_{1}{\zeta }_{{m}_{I}-1\leftrightarrow {m}_{I}}^{{{{{{\rm{NMR}}}}}}}$
${f}_{{m}_{I}-1\leftrightarrow {m}_{I}}^{{{{{{\rm{NM}}}}}}{{{{{{\rm{R}}}}}}}^{0}}={\gamma }_{{{{{{\rm{n}}}}}}}{B}_{0}+\left({m}_{I}-\frac{1}{2}\right){f}_{{{{{{\rm{q}}}}}}}^{0}\pm \frac{A}{2}+O({A}^{2})$	${\zeta }_{{m}_{I}-1\leftrightarrow {m}_{I}}^{{{{{{\rm{NMR}}}}}}}$	${f}_{{m}_{I}-1\leftrightarrow {m}_{I}}^{{{{{{\rm{Rabi}}}}}},{{{{{\rm{NM}}}}}}{{{{{{\rm{R}}}}}}}^{0}}={f}_{{m}_{I}-1\leftrightarrow {m}_{I}}^{{{{{{\rm{Rabi}}}}}},{{{{{\rm{NM}}}}}}{{{{{{\rm{R}}}}}}}^{+}}\left(\frac{A}{2{B}_{0}{\gamma }_{{{{{{\rm{n}}}}}}}}\pm 1\right)$
${f}_{{m}_{I}-1\leftrightarrow {m}_{I}}^{{{{{{\rm{NE}}}}}}{{{{{{\rm{R}}}}}}}^{+}}={f}_{{m}_{I}-1\leftrightarrow {m}_{I}}^{{{{{{\rm{NM}}}}}}{{{{{{\rm{R}}}}}}}^{+}}={\gamma }_{{{{{{\rm{n}}}}}}}{B}_{0}+\left({m}_{I}-\frac{1}{2}\right){f}_{{{{{{\rm{q}}}}}}}^{+}$	${\alpha }_{{m}_{I}-1\leftrightarrow {m}_{I}}^{{{{{{\rm{NER}}}}}}}=\frac{\left|2{m}_{I}-1\right|}{2}\sqrt{I\left(I+1\right)-{m}_{I}({m}_{I}-1)}$	${f}_{{m}_{I}-1\leftrightarrow {m}_{I}}^{{{{{{\rm{Rabi}}}}}},{{{{{\rm{NE}}}}}}{{{{{{\rm{R}}}}}}}^{+}}={\alpha }_{{m}_{I}-1\leftrightarrow {m}_{I}}^{{{{{{\rm{NER}}}}}}}\sqrt{\delta {Q}_{{xz}}^{2}+\delta {Q}_{{yz}}^{2}}$
${f}_{{m}_{I}-2\leftrightarrow {m}_{I}}^{{{{{{\rm{NE}}}}}}{{{{{{\rm{R}}}}}}}^{+}}={f}_{{m}_{I}-1\leftrightarrow {m}_{I}}^{{{{{{\rm{NE}}}}}}{{{{{{\rm{R}}}}}}}^{+}}$+${f}_{{m}_{I}-2\leftrightarrow {m}_{I}-1}^{{{{{{\rm{NE}}}}}}{{{{{{\rm{R}}}}}}}^{+}}$	${\beta }_{{m}_{I}-2\leftrightarrow {m}_{I}}^{{{{{{\rm{NER}}}}}}}=\frac{1}{4}\sqrt{\left(I-{m}_{I}-7\right)\left(I-{m}_{I}-6\right)\left(I-{m}_{I}+1\right)\left(I-{m}_{I}+2\right)}$	${f}_{{m}_{I}-2\leftrightarrow {m}_{I}}^{{{{{{\rm{Rabi}}}}}},{{{{{\rm{NE}}}}}}{{{{{{\rm{R}}}}}}}^{+}}={\beta }_{{m}_{I}-2\leftrightarrow {m}_{I}}^{{{{{{\rm{NE}}}}}}{{{{{{\rm{R}}}}}}}^{+}}\sqrt{{\left(\delta {Q}_{{xx}}-\delta {Q}_{{yy}}\right)}^{2}+4\delta {Q}_{{xy}}^{2}}$
${f}_{{m}_{I}-1\leftrightarrow {m}_{I}}^{{{{{{\rm{EDSR}}}}}}}={\gamma }_{+}{B}_{0}+\left({m}_{I}-\frac{1}{2}\right)\left({f}_{{{{{{\rm{q}}}}}}}^{0}+A\right)$	${\delta }_{{m}_{I}-1\leftrightarrow {m}_{I}}^{{{{{{\rm{EDSR}}}}}}}={\zeta }_{{m}_{I}-1\leftrightarrow {m}_{I}}^{{{{{{\rm{NMR}}}}}}}$	${f}_{{m}_{I}-1\leftrightarrow {m}_{I}}^{{{{{{\rm{Rabi}}}}}},{{{{{\rm{EDSR}}}}}}}=\Delta A{\delta }_{{m}_{I}-1\leftrightarrow {m}_{I}}^{{{{{{\rm{EDSR}}}}}}}$
${f}_{{m}_{I}}^{{{{{{\rm{ESR}}}}}}}={\gamma }_{{{{{{\rm{e}}}}}}}{B}_{0}+{m}_{I}A+O({A}^{2})$	$-$	${f}_{{m}_{I}}^{{{{{{\rm{Rabi}}}}}},{{{{{\rm{ESR}}}}}}}=\frac{{\gamma }_{{{{{{\rm{e}}}}}}}{B}_{1}}{2}$

Here, *I* = 7/2, *m*_*I*_ = { − *I*, − *I* + 1. . . , *I*} and *γ*_+_ = *γ*_n_ + *γ*_e_, where *γ*_n_ = 5.55 MHz and *γ*_e_ = 27.97 GHz. The notation employed for ${f}_{{m}_{I}-1\leftrightarrow {m}_{I}}^{{{{{{{{\rm{NM{R}}}}}}}^{0}}}}$ assigns the positive sign (+) and negative sign (-) to the resonance frequency when the electron is in the spin-down or spin-up state, respectively.

To manipulate and read out the 16-dimensional Hilbert space of the single ^123^Sb, we use a silicon nanoelectronic device as shown in Fig. 1c (fabrication details in Supplementary Section 2). The device features a single electron transistor (SET) to read out the spin of the donor-bound electron^40^, a set of gates to control the electrostatic potential of the donor or drive NER^24^, and a broadband short-circuited microwave antenna used to deliver the *B*_1_ field for ESR and NMR. To drive the donor spins electrically at microwave frequencies via EDSR, we exploit the stray electric fields from the microwave antenna.

### Resonance spectra and energy level addressability

The spin resonance spectrum of the ionised nucleus is reported in Fig. 1d (${\rm{NMR}}_{\pm 1}^{+}$) and Fig. 1g (${\rm{NER}}_{\pm 1}^{+}$). The spectra are of course identical, except for the absence of the *m*_*I*_ = − 1/2 ↔ + 1/2 transition in the ${\rm{NER}}_{\pm 1}^{+}$ case, due to the selection rules imposed by modulation of the quadrupole interaction^24^. The static quadrupole splitting ${f}_{{{{{{{{\rm{q}}}}}}}}}^{+}=-44.1(2)$ kHz (here and elsewhere, error bars indicate 1*σ* standard deviations) is obtained directly from the distance between adjacent peaks. The presence of a nonzero quadrupole splitting ensures that all pairs of nuclear levels are individually addressable, as required for complete SU(8) control of the qudit^1^. We know the sign of ${f}_{{{{{{{{\rm{q}}}}}}}}}^{+}$ because we are able to deterministically initialise a specific nuclear state $\left\vert {m}_{I}\right\rangle$ through a combination of ESR and EDSR transitions (see Supplementary Materials, Section 3) and thus identify the $\left\vert 7/2\right\rangle \leftrightarrow \left\vert 5/2\right\rangle$ transition as the one at the lowest frequency. The numerical value of ${f}_{{{{{{{{\rm{q}}}}}}}}}^{+}$ is close to that observed in a similar device^24^ and is well understood as arising from the EFG produced by static strain in the device as a consequence of the differential thermal expansion of the aluminium gates placed over the silicon^24,41^.

The NMR frequency for *m*_*I*_ = − 1/2 ↔ + 1/2 is equal to the Zeeman splitting *γ*_n_*B*_0_, without contributions from the quadrupole interaction. This allows us to accurately calibrate the static magnetic field value, *B*_0_ = 999.5(5) mT, which is provided by an array of permanent magnets^42^ and thus not precisely known a priori.

When the donor is in the charge-neutral state, the ${\rm{NMR}}_{\pm 1}^{0}$ frequencies are shifted equally to first order by the hyperfine interaction, and further split by second-order hyperfine terms *O*(*A*^2^) ∝ *A*^2^/*γ*_e_*B*_0_, depending on the nuclear spin projection (see Supplementary Materials, Section 4). This can be appreciated in Fig. 1e where the frequency axis has been offset by the linear contribution of the hyperfine coupling *A*/2, which is equal for all the transitions. Plotting the ${\rm{NMR}}_{\pm 1}^{+}$ (Fig. 1d) and the ${\rm{NMR}}_{\pm 1}^{0}$ (Fig. 1e) spectra across the same frequency spread ≈ ± 1 MHz highlights that, in the neutral case, the splitting caused by the *O*(*A*^2^) terms is much larger than ${f}_{{{{{{{{\rm{q}}}}}}}}}^{+}$, proving that all ${\rm{NMR}}_{\pm 1}^{0}$ transitions would be individually addressable even in the absence of quadrupole effects. From the ${\rm{NMR}}_{\pm 1}^{0}$ spectrum we extract *A* = 96.584(2) MHz and ${f}_{{{{{{{{\rm{q}}}}}}}}}^{0}=-52.5(5)$ kHz (see Supplementary Materials, Section 4 for calculation details). The quadrupole splitting thus differs by ≈ 8 kHz between the neutral and the ionised donor case. This could be due to a small additional EFG contribution from the electron wavefunction, which is itself distorted from its 1*s* symmetry by the local strain^35^.

The eight ESR resonances (Fig. 1f), each conditional on one of the *m*_*I*_ nuclear spin projections, are split by the hyperfine interaction $A\hat{{{{{{{{\boldsymbol{S}}}}}}}}}\cdot \hat{{{{{{{{\boldsymbol{I}}}}}}}}}$. A detailed calculation (see Supplementary Materials, Section 5) shows that both first- and second-order terms in *A* contribute to the ESR frequency splitting, whereas only the resonances conditional on *m*_*I*_ = ± 1/2 are separated by exactly *A*. We also observe the seven expected EDSR flip-flop transitions (Fig. 1i), where both the electron and nucleus undergo simultaneous spin flips with Δ(*m*_*I*_ + *m*_*S*_) = 0, driven by the electrical modulation of the hyperfine interaction.

### Coherent nuclear spin control

**Fig. 2:**
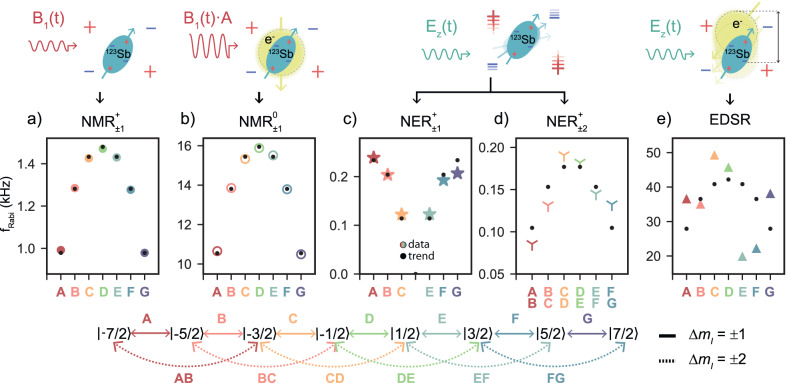
Coherent magnetic and electric drive of the ^123^Sb nuclear spin. **a** Rabi trends obtained when driving the ionised nucleus with an oscillating magnetic field, through NMR. **b** Trends for NMR on the charge-neutral atom, with the electron in the spin-down state. The hyperfine-enhanced nuclear gyromagnetic ratio yields faster Rabi oscillations for the same *B*_1_ amplitude. The experiments in panels **a**, **b** were carried out by applying a voltage of ${V}_{{{{{{{{\rm{RF}}}}}}}}}^{{{{{{{{\rm{pp}}}}}}}}}=50$ mV to the input of the on-chip antenna. This is calculated by accounting solely for the effect of a 10 dB attenuator at the 4 K stage, since line losses are negligible at NMR frequencies. **c**, **d** Rabi frequencies obtained by driving the nucleus via NER, through the electrical modulation of the quadrupolar interaction for **c** Δ*m* = 1, and **d** Δ*m* = 2. In both cases, this is achieved by applying an oscillating voltage with an amplitude of ${V}_{{{{{{{{\rm{RF}}}}}}}}}^{{{{{{{{\rm{pp}}}}}}}}}=60$ mV to a donor gate. **e** Stray electric fields from the microwave antenna (–6 dBm at source) are used to drive electron-nuclear spin transitions coherently (through EDSR). The physical mechanisms that drive the nuclear spins are illustrated above each panel. We label and colour code the nuclear spin transitions using the diagram below the panels.

Having identified all the resonance frequencies of the ^123^Sb system, we demonstrate five different methods of driving coherent rotations on the nuclear spin qudit, including NMR for the ionised (${\rm{NMR}}_{\pm 1}^{+}$) and neutral (${\rm{NMR}}_{\pm 1}^{0}$) atom, ionised ${\rm{NER}}_{\pm 1,\pm 2}^{+}$, and EDSR (Fig. 2). A notable feature of magnetic and electric drive in high-spin systems is the dependence of the Rabi frequencies on the nuclear spin number *m*_*I*_, which arises from the distinct transition matrix elements in the driving operators^24^. Table 1 summarises the nuclear-spin-dependent scaling coefficients and driving amplitudes for the different driving mechanisms.

With magnetic (NMR) drive, the oscillating magnetic field ${B}_{1}\cos (2\pi {f}_{{m}_{I}-1\leftrightarrow {m}_{I}}^{{{{{{{{\rm{NMR}}}}}}}}}t)$ couples to the nuclear spin via the off-diagonal matrix elements of the ${\hat{I}}_{x}$ spin operator. Therefore, the Rabi rates are expected to increase for smaller ∣*m*_*I*_∣, in both the ionised and neutral case, as observed in the data in Fig. 2a, b. We find the neutral donor Rabi rates to be enhanced with respect to the ionised case by a factor ${f}_{{{{{{{{\rm{Rabi}}}}}}}}}^{{{{{{{{\rm{NMR0}}}}}}}}}/{f}_{{{{{{{{\rm{Rabi}}}}}}}}}^{{{{{{{{\rm{NMR+}}}}}}}}}=10.776(8)$, which is consistent with a hyperfine-enhanced nuclear gyromagnetic ratio^43^. This is a consequence of electron-nuclear state mixing through the transverse term of the hyperfine interaction, $A{\hat{S}}_{x}{\hat{I}}_{x}$, which effectively creates an additional driving field of magnitude $\frac{A{B}_{1}}{2{\gamma }_{{{{{{{{\rm{n}}}}}}}}}{B}_{0}}$ along the *x-*axis, adding to the external *B*_1_. Using the measured values of *A* and *B*_0_, this mechanism predicts an increase in Rabi rates ${f}_{{{{{{{{\rm{Rabi}}}}}}}}}^{{{{{{{{\rm{NM{R}}}}}}}^{0}}}}/{f}_{{{{{{{{\rm{Rabi}}}}}}}}}^{{{{{{{{\rm{NM{R}}}}}}}^{+}}}}=(1+\frac{A}{2{\gamma }_{{{{{{{{\rm{n}}}}}}}}}{B}_{0}})\, \approx \,9.6$. The slight discrepancy with the measured enhancement is likely due to a different frequency response of the driving circuitry at ${f}^{{{{{{{{\rm{NM{R}}}}}}}^{+}}}}\approx 5.5$ MHz and ${f}^{{{{{{{{\rm{NM{R}}}}}}}^{0}}}}\approx 54$ MHz.

For electrical drive with Δ*m*_*I*_ = ± 1 (${\rm{NER}}_{\pm 1}^{+}$), the relevant transition matrix elements come from the quadrupolar interaction involving the operators: ${\hat{I}}_{x}{\hat{I}}_{z}$, ${\hat{I}}_{z}{\hat{I}}_{x}$, ${\hat{I}}_{y}{\hat{I}}_{z}$, ${\hat{I}}_{z}{\hat{I}}_{y}$. The fastest Rabi rates in this case are found at larger ∣*m*_*I*_∣, whereas the $\left\vert -1/2\right\rangle \leftrightarrow \left\vert 1/2\right\rangle$ transition is completely forbidden. This behaviour is reflected in Fig. 2c, showing the expected decreasing Rabi rates for lower ∣*m*_*I*_∣, and the missing value for the middle transition. The ‘double transition’ ${\rm{NER}}_{\pm 2}^{+}$ is obtained by modulating quadratic terms of the form ${\hat{I}}_{\alpha }{\hat{I}}_{\alpha }$ with *α*, *β* ∈ {*x*, *y*} whose matrix elements are larger for lower ∣*m*_*I*_∣, thus similar to NMR. This is confirmed by the data in Fig. 2d.

The nuclear spin can be driven electrically at microwave frequencies via EDSR, through the modulation of the hyperfine interaction^38,44^. In this case, the trends are expected to match those obtained for NMR. We use the stray electric fields from the microwave antenna to drive EDSR, and extract the Rabi frequencies for all the flip-flop transitions (Fig. 2e). Here, the measured Rabi frequencies show no clear trend because of the strongly frequency-dependent response of the microwave antenna in the range of ${f}_{{m}_{I}-1\leftrightarrow {m}_{I}}^{{{{{{{{\rm{EDSR}}}}}}}}}\approx 28$ GHz. This is also evident in the different Rabi frequencies obtained for ESR (see Supplementary Materials, Section 6), where no dependence in nuclear spin number is expected (Table 1). The observed ${f}_{5/2\leftrightarrow 7/2}^{{{{{{{{\rm{Rabi,EDSR}}}}}}}}}/{\delta }_{5/2\leftrightarrow 7/2}^{{{{{{{{\rm{EDSR}}}}}}}}}\approx 28$ kHz is obtained using ${V}_{{{{{{{{\rm{MW}}}}}}}}}^{{{{{{{{\rm{pp}}}}}}}}}=300$ mV of driving amplitude at the source, which corresponds to ${V}_{{{{{{{{\rm{MW}}}}}}}}}^{{{{{{{{\rm{pp}}}}}}}}}\, \approx \, 30$ mV at the input of the antenna. The total attenuation along the transmission line is ≈ 20 dB at ≈ 28 GHz, as the combined effect of a 10 dB attenuator installed at the 4 K plate, and another ≈ 10 dB of loss along the coaxial cable at these frequencies^38^. In a device with ^31^P donors and a dedicated open-circuited antenna to deliver microwave electric fields, a similar value of ${f}_{{{{{{{{\rm{Rabi}}}}}}}}}^{{{{{{{{\rm{EDSR}}}}}}}}}$ required ${V}_{{{{{{{{\rm{MW}}}}}}}}}^{{{{{{{{\rm{pp}}}}}}}}}=3$ V^38^ at the source, with the same line attenuation as in the setup used here. As we discuss below, this is an indication that the hyperfine Stark shift in ^123^Sb is much larger than in ^31^P.

### Electrical tunability of the resonance frequencies

The ^123^Sb Hamiltonians, Eqs. ((1), (2)), contain terms that depend on the electric field applied to the donor, which itself depends on the DC voltages applied to the gates, *V*_DC_. For the ionised donor, the only electrically-tunable term is the nuclear quadrupole interaction, which depends on the applied voltage through the linear quadrupole Stark effect (LQSE)^24,45^. The shift of the ${\rm{NMR}}_{\pm 1}^{+}$ resonance as a function of the DC voltage on donor gate 1, ${V}_{{{{{{{{\rm{DC}}}}}}}}}^{{{{{{{{\rm{DG1}}}}}}}}}$, obeys the relation

3 $$\Delta {f}_{{m}_{I}-1\leftrightarrow {m}_{I}}^{{{{{{{{{\rm{NMR}}}}}}}}}^{+}}=\left({m}_{I}-\frac{1}{2}\right)\Delta {f}_{{{{{{{{\rm{q}}}}}}}}}^{+},$$

where $\Delta {f}_{{{{{{{{\rm{q}}}}}}}}}^{+}=(\partial {f}_{{{{{{{{\rm{q}}}}}}}}}^{+}/\partial V)\cdot \Delta {V}_{{{{{{{{\rm{DC}}}}}}}}}^{{{{{{{{\rm{DG1}}}}}}}}}$. In this device, we measure $\partial {f}_{{{{{{{{\rm{q}}}}}}}}}^{+}/\partial V=-2.07(2)\,{{{{{{{\rm{kHz/V}}}}}}}}$ (Supplementary Materials, Section 7C).

**Fig. 3:**
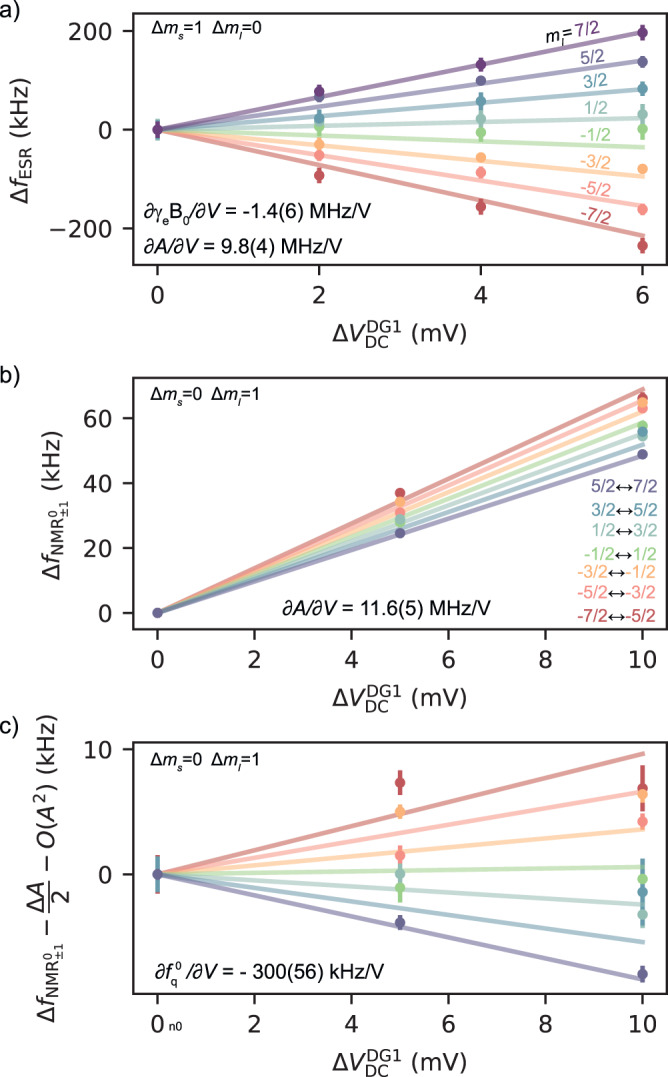
Stark effect. **a** Stark shift on the ESR resonance frequencies as a function of gate voltage variation, denoted by Δ${V}_{{{{{{{{\rm{DC}}}}}}}}}^{{{{{{{{\rm{DG1}}}}}}}}}$, for all nuclear spin projections *m*_*I*_. **b** Stark shift on the neutral NMR resonance frequencies as a function of Δ${V}_{{{{{{{{\rm{DC}}}}}}}}}^{{{{{{{{\rm{DG1}}}}}}}}}$, for all nuclear spin transitions $\left\vert \downarrow,{m}_{I}-1\right\rangle \leftrightarrow \left\vert \downarrow,{m}_{I}\right\rangle$. **c** The NMR Stark shift when subtracting the linear and second-order hyperfine contributions is shown to highlight the nuclear-dependent trends arising from the LQSE. The solid lines in all panels are obtained numerically by solving Eq. (2) as a function of ${V}_{{{{{{{{\rm{DC}}}}}}}}}^{{{{{{{{\rm{DG1}}}}}}}}}$ using the experimentally obtained Stark effect parameters.

In the neutral donor, electric fields additionally affect the electron gyromagnetic ratio *γ*_e_ and the hyperfine coupling *A* through the Stark effect^26,39^. The ESR frequency shifts as a function of gate voltage as:

4 $$\Delta {f}_{{{{{{{{\rm{ESR}}}}}}}}}=\Delta {\gamma }_{{{{{{{{\rm{e}}}}}}}}}{B}_{0}+2{m}_{I}\Delta A,$$

where Δ*γ*_e_ and Δ*A* describe a change in the coupling parameters as a function of ${V}_{{{{{{{{\rm{DC}}}}}}}}}^{{{{{{{{\rm{DG1}}}}}}}}}$. The factor *m*_*I*_ indicates that the eight ESR frequencies shift at different rates for a change in *A*, whereas a change in *γ*_e_ causes all frequencies to move by the same amount. The clear fan-out of the ESR frequencies in Fig. 3a shows that the hyperfine Stark shift is the dominant effect here. A fit to the data yields ∂*γ*_e_*B*_0_/∂*V* = − 1.4(6) MHz/V and ∂*A*/∂*V* = 9.8(4) MHz/V (See Supplementary Materials, Section 7A). The hyperfine Stark shift is a factor ≈ 10 larger than was observed in a ^31^P donor device^26^. A similar enhancement, albeit for the quadratic Stark effect, was found with multi-valley effective mass models and experiments conducted on bulk donors in silicon^39^. The larger hyperfine Stark shift compared to ^31^P results in a faster driving of the electron-nuclear flip-flop transition. Indeed, here we were able to coherently drive the flip-flop transitions using just with the stray electric field generated at the ESR antenna (nominally optimised for delivering oscillating magnetic fields), and do so even more efficiently than in a ^31^P device with a dedicated open-circuit electrical antenna^38^. Furthermore, we verify that the donor under study operates in a regime where the hyperfine Stark shift is linear in voltage (Supplementary Materials, Section 8).

The neutral ${\rm{NMR}}_{\pm 1}^{0}$ frequencies are voltage-dependent through the hyperfine Stark shift Δ*A* and the LQSE $\Delta {f}_{{{{{{{{\rm{q}}}}}}}}}^{0}$:

5 $$\Delta {f}_{{m}_{I}-1\leftrightarrow {m}_{I}}^{{{{{{{{\rm{NM{R}}}}}}}^{0}}}}=	\left({m}_{I}-\frac{1}{2}\right)\Delta {f}_{{{{{{{{\rm{q}}}}}}}}}^{0}\pm \frac{1}{2}\Delta A\\ 	+{g}_{{m}_{I}-1\leftrightarrow {m}_{I}}\frac{2A}{{\gamma }_{{{{{{{{\rm{e}}}}}}}}}{B}_{0}}\Delta A,$$

where the last term corresponds to second-order corrections to the hyperfine interaction, which are comparable in magnitude to the LQSE. The factor (*m*_*I*_ − 1/2) preceding $\Delta {f}_{{{{{{{{\rm{q}}}}}}}}}^{0}$ and the coefficient ${g}_{{m}_{I}-1\leftrightarrow {m}_{I}}$ are now responsible for making $\Delta {f}_{{m}_{I}-1\leftrightarrow {m}_{I}}^{{{{{{{{\rm{NM{R}}}}}}}^{0}}}}$ depend on the nuclear spin transition. From the data in Fig. 3c we extract $\partial {f}_{{{{{{{{\rm{q}}}}}}}}}^{0}/\partial V=-300(56)$ kHz/V and ∂*A*/∂*V* = 11.57(45) MHz/V (Supplementary Materials, Section 7B). The slight difference between the estimated ∂*A*/∂*V* extracted from the data in Fig. 3a, b may be attributed to variations in the DC voltage settings between measurements, potentially impacting the electron’s wavefunction sensitivity to electric fields^44^.

Because the shift in resonance frequencies is dominated by Δ*A*/2, in Fig. 3c we plot $\Delta {f}_{{m}_{I}-1\leftrightarrow {m}_{I}}^{{{{{{{{\rm{NM{R}}}}}}}^{0}}}}-\Delta A/2-O({A}^{2})$ to highlight the contribution of the LQSE to the nuclear spin dependent fan-out (Fig. 3c). Notably, the value obtained for LQSE in the ionised nucleus is two orders of magnitude smaller than the one obtained for the neutral atom. This observation could be used in the future to refine and validate ab initio models of the nuclear quadrupole interaction.

### Decoherence: magnetic and electric noise

The key property of ^31^P donor qubits is their exceptionally long coherence times^16^, largely due to their weak sensitivity to electric fields. The ionised nucleus is strictly unaffected by electric fields due to its spin *I* = 1/2. Moving to a heavier donor like ^123^Sb, with larger hyperfine Stark shifts and a nuclear electric quadrupole moment, raises the question of whether this will deteriorate spin coherence.

**Fig. 4:**
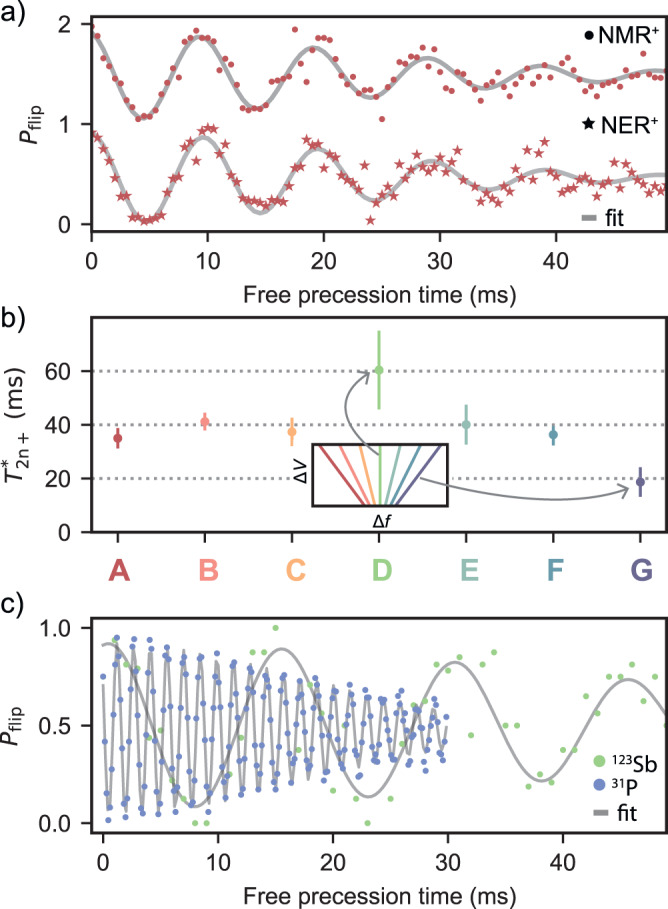
Electric and magnetic noise on the ionised nucleus. **a** Ramsey decay for the transition $\left\vert -7/2\right\rangle \leftrightarrow \left\vert -5/2\right\rangle$ using NMR and NER. ${T}_{2{{{{{{{\rm{n+}}}}}}}}}^{*}\approx 29$ ms in both cases, indicating no effect of the driving mechanism on the dephasing rates. **b** Dephasing times ${T}_{2{{{{{{{\rm{n+}}}}}}}}}^{*}$ measured with a Ramsey sequence for all *m*_*I*_, showing an increased ${T}_{2{{{{{{{\rm{n+}}}}}}}}}^{*}$ for the $\left\vert -1/2\right\rangle \leftrightarrow \left\vert 1/2\right\rangle$ transition. The duration of the Ramsey experiments lasted for a period of 3 hours. The inset depicts the linear quadrupole Stark effect on the resonance frequencies, to illustrate that the inner transition is unaffected by electric fields. The large error bar for D is attributed to a lower fidelity in state preparation (Supplementary Materials, section 1).The elevated nuclear flipping rates for $\left\vert -1/2\right\rangle \leftrightarrow \left\vert 1/2\right\rangle$ led to more discarded individual runs during the Ramsey experiment, as the measured data points frequently fell outside the desired nuclear subspace. This led to a reduced sampling of this subspace, resulting in higher statistical errors in the fitted decoherence times. **c** Superimposed Ramsey decays for ionised ^31^P and ^123^Sb nuclei, both measured on the electric-field insensitive $\left\vert -1/2\right\rangle \leftrightarrow \left\vert 1/2\right\rangle$ transitions, showing a shorter ${T}_{2}^{*}$ for the ^31^P nucleus, in proportion to its larger gyromagnetic ratio.

Focussing on the ionised nucleus, we first verify that the driving mechanism does not affect the dephasing time ${T}_{{{{{{{{\rm{2n+}}}}}}}}}^{*}$. Figure 4a compares two Ramsey experiments on the $\left\vert -7/2\right\rangle \leftrightarrow \left\vert -5/2\right\rangle$ transition where the *π*/2 pulses were delivered using either NMR or NER. We found near-identical values ${T}_{{{{{{{{\rm{2n+}}}}}}}}}^{*}=29.4(3)$ ms with NMR and ${T}_{{{{{{{{\rm{2n+}}}}}}}}}^{*}=29.8(3)$ ms with NER. This is intuitively expected because the Ramsey experiment probes the free evolution of the spin, in the absence of drives. However, this result indicates that the application of strong AC electric fields needed to drive NER does not destabilise the electrical environment of the nucleus in a noticeable way^46^.

The ionised ^123^Sb nucleus offers a unique opportunity to rigorously distinguish magnetic from electric contributions to the noise that affects the spin coherence. Since ${f}_{{m}_{I}-1\leftrightarrow {m}_{I}}^{{{{{{{{\rm{NM{R}}}}}}}^{+}}}}={\gamma }_{n}{B}_{0}+({m}_{I}-1/2){f}_{{{{{{{{\rm{q}}}}}}}}}^{+}$, quadrupole shifts caused by electric fields do not affect the coherence of the $\left\vert -1/2\right\rangle \leftrightarrow \left\vert 1/2\right\rangle$ transition^32^, i.e., the spin-1/2 nuclear subspace behaves (to first order) like a ^31^P donor nucleus (*I* = 1/2) would. Figure 4b shows the dephasing times ${T}_{{{{{{{{\rm{2n+}}}}}}}}}^{*}$ as a function of *m*_*I*_ (Fig. 4b) for all transitions, measured using NMR. The $\left\vert -1/2\right\rangle \leftrightarrow \left\vert 1/2\right\rangle$ transition has a ≈ 1.5 × longer coherence than the outer transitions. The ionised ^123^Sb nucleus thus couples measurably to electric-field noise, but the coherence degradation is only by a factor of order unity in this type of devices, despite the fact that decoherence channels of magnetic origin are already minimised by the use of an isotopically purified ^28^Si substrate. By comparison, a factor ~ 10 degradation in ${T}_{2}^{{{{{{{{\rm{H}}}}}}}}}$ between the inner and the outer transitions was observed in experiments on ensembles of near-surface As^+^ donors in natural Si^32^, indicating that the electrical and charge noise level in our devices is remarkably benign.

In this particular device we co-implanted a small dose of ^31^P donors, and we were able to address one of them. This allowed us to measure the dephasing time of two different donor species in the same device (Fig. 4c). The ionised ^31^P donor nucleus has only one NMR transition, $\left\vert -1/2\right\rangle \leftrightarrow \left\vert 1/2\right\rangle$, for which we found ${T}_{{{{{{{{\rm{2n+P}}}}}}}}}^{*}=24.5(5)$ ms. Taking the ratio of ${T}_{{{{{{{{\rm{2n+}}}}}}}}}^{*}$ for the same transition in ^123^Sb yields ${T}_{2n+{{{{{{{\rm{Sb}}}}}}}}}^{*}/{T}_{2n+{{{{{{{\rm{P}}}}}}}}}^{*}=2.5(6)$, in agreement with the ratio of the nuclear gyromagnetic ratios *γ*_n,P_/*γ*_n,Sb_ = 3.1, where *γ*_n,P_ = 17.23 MHz/T. A small discrepancy could be caused by a different distribution of residual ^29^Si spins around each donor. Additional data on relaxation and coherence times is discussed in the Supplementary Materials, Sections 9–10.

### Gate fidelities

In preparation for future work on qudits^1^ and logical qubits^30^ encoding on the ^123^Sb system, we used gate set tomography (GST)^17,47^ to benchmark the performance of one-qubit gates. We chose the qubit basis as the $\left\vert 0\right\rangle=\left\vert -5/2\right\rangle$ and $\left\vert 1\right\rangle=\left\vert -7/2\right\rangle$ states of the ionised donor nucleus, and assessed the performance of the *X*_*π*/2_,*Y*_*π*/2_ and ${\mathbb{I}}$ gates, for both magnetic (${\rm{NMR}}_{\pm 1}^{+}$) and electric (${\rm{NER}}_{\pm 1}^{+}$) drive. The *X*_*π*/2_ and *Y*_*π*/2_ gates represent half rotations of the spin around the Bloch sphere, achieved through simple rectangular-envelope pulses modulating an oscillating driving field in resonance with the qubit Larmor frequency. The idle gate ${\mathbb{I}}$ employs a far off-resonance stimulus that does not drive the qubit but delivers the same power to the device as the other gates. This helps reduce context-dependent errors, where the frequency of the qubit or the readout contrast in the charge sensor is affected by the presence or absence of a driving field^38,48^. The results are presented in Table 2 and show that all driven gates have average fidelity higher than 99.3% (see Supplementary Materials, Section 11, for details on the error generators).

**Table 2:** Gate set tomography results

Gate	Pulse duration		Average gate fidelity	
	NMR	NER	NMR	NER
${\mathbb{I}}$	255 μs	1.2087 ms	99.42 (30)%	98.35 (44)%
*X*_*π*/2_	255 μs	1.2087 ms	99.88 (25)%	99.76 (26)%
*Y*_*π*/2_	255 μs	1.2087 ms	99.82 (24)%	99.96 (27)%

## Discussion

We have presented the experimental demonstration of coherent control of the electron and nuclear states of a single ^123^Sb donor atom, ion-implanted in a silicon nanoelectronic device. The combined Hilbert space of the atom spans 16 dimensions, and can be accessed using both electric and magnetic control fields. The exquisite spectral resolution afforded by the weak spin decoherence allowed us to extract detailed information on the value and the tunability of the Hamiltonian terms that determine the atom’s quantum behaviour. The nuclear spin already shows gate fidelities exceeding 99% regardless of the drive mechanism. Further improvements to coherence times and gate fidelities are likely to become possible in the near future by adopting isotopically purified ^28^Si substrates with much-reduced residual ^29^Si concentration. Recent experiments have achieved exceptionally low 2.3(7) ppm residual ^29^Si by a focused ion beam enrichment method^49^, which we will seek to introduce within our process flow in the near future.

The ^123^Sb donor also presents advantages in the effort of scaling up to large-scale quantum processors. ^123^Sb is much heavier than ^31^P, which results in a much-reduced implantation straggle. At the same implantation energy, the ion-induced charge signal, used for the deterministic implantation of single donors, is larger in ^123^Sb compared to ^31^P. Recent work demonstrated >99.99% confidence in counting a single ^123^Sb^+^ ion at 18 keV implantation energy, and the deterministic formation of a 16 × 16 array of Sb donors^50^.

Future work will focus on exploiting the large Hilbert space for the creation of Schrödinger cat states^51^. These may have application in quantum sensing^52^, to beat the standard quantum limit of phase estimation, and in quantum foundations, to prove that the simple probing of a spin precession can be sufficient to detect quantumness^53^. Another quantum foundations experiment afforded by the large Hilbert space is the test of the reality of the quantum state, where the bound on the inadequacy of a purely epistemic view becomes tighter in higher dimensions^54^. The relation between lattice strain and nuclear quadrupole interaction will be exploited to demonstrate nuclear acoustic resonance^25^, and to use the ^123^Sb atom as a local probe for strain in semiconductor nanoscale devices^55^. For quantum information processing, an exciting prospect is the encoding of an error-correctable logical qubit in the *I* = 7/2 nuclear spin^30^. Multiple nuclei could be further entangled using the same electron-mediated two-qubit gates already demonstrated in ^31^P^17^, which require two nuclei to be placed at a distance ≈ 5 nm in order to share a common hyperfine-coupled electron. The large implantation straggling of ^31^P precludes the deterministic formation of such closely-spaced donor pairs, which are only found fortuitously. Conversely, pairs of ^123^Sb donor spaced by ≈ 5 nm can be obtained deterministically by implanting ${\rm{Sb}}_{2}^{+}$ diatomic molecules. Upon impact with the surface, the atoms in the molecule break apart, and the kinetics of the ion stopping in the substrate lands the two atoms at ≈ 5 nm distance with a high probability^50^. Finally, the high tunability of the hyperfine coupling observed in our experiment bodes well for the prospect of using electric-dipole coupling in a flip-flop qubit architecture^44^, which is ideally suited for donor arrays with ≈ 200 nm pitch, such as the ones recently fabricated using deterministic ion implantation^50^. It may also facilitate the implementation of control protocols where individual atoms are brought in and out of resonance with global oscillating magnetic fields using localised electrical control^26,56^.

### Supplementary information

Supplementary information

Peer Review File

## Data Availability

All data needed to evaluate the conclusions in the paper are present in the paper and/or the Supplementary Materials. All the data and analysis scripts supporting the contents of the manuscript can be downloaded from the following repository: https://datadryad.org/stash/share/yypCpKkL1wniO7Il3EFKbSDYfDka9qIcQznHX5ssyvs.
