# Supplementary material for: Navigating the 16-dimensional Hilbert space of a high-spin donor qudit with electric and magnetic fields
Source: Nat Commun. 2024 Feb 14;15:1380. doi: 10.1038/s41467-024-45368-y (PMC11258329; doi:10.1038/s41467-024-45368-y)
Supplement: Supplementary file 1 — Supplementary information [file 41467_2024_45368_MOESM1_ESM.pdf]

Supplementary Information: Navigating the 16-dimensional Hilbert space of a high-spin donor qudit with electric and magnetic fields

Irene Fernández de Fuentes,[1] Tim Botzem,[1] Mark A. I. Johnson,[1] Arjen Vaartjes,[1] Serwan Asaad,[1] Vincent Mourik,[1] Fay. E. Hudson,[1,2] Kohei M. Itoh,[3] Brett C. Johnson,[4] Alexander M. Jakob,[5] Jeffrey C. McCallum,[5] David N. Jamieson,[5] Andrew S. Dzurak,[1,2] and Andrea Morello[1,*]

[1]*School of Electrical Engineering and Telecommunication, UNSW Sydney, Sydney, New South Wales, Australia*

[2]*Diraq, Sydney, New South Wales, Australia*

[3]*School of Fundamental Science and Technology, Keio University, Yokohama, Japan*

[4]*School of Science, RMIT University, Melbourne, Victoria, Australia*

[5]*School of Physics, University of Melbourne, Melbourne, Victoria, Australia*

CONTENTS

Supplementary Note 1: Random nuclear spin flips / ionisation shock S2

Supplementary Note 2: Device fabrication and operation S3

Supplementary Note 3: Nuclear state preparation for ^{123}Sb S4

Supplementary Note 4: Calculation of quadrupolar splitting for $\mathrm{NMR}^{0}_{\pm1}$ S5

Supplementary Note 5: Calculation of hyperfine interaction from ESR spectrum S6

Supplementary Note 6: Rabi oscillations S7

Supplementary Note 7: Calculation of the Stark effect S11
A. Electron spin S11
B. Nuclear spin (neutral donor) S11
C. Nuclear spin (ionised donor) S12

Supplementary Note 8: Quadratic nuclear Stark effect S12

Supplementary Note 9: Electron T_1 time S14

Supplementary Note 10: Coherence times S14

Supplementary Note 11: GST Experiments S16

Supplementary Note 12: Voltage and frequency fluctuations with laboratory temperature S18

Supplementary References S20

* Corresponding author: a.morello@unsw.edu.au

SUPPLEMENTARY NOTE 1: RANDOM NUCLEAR SPIN FLIPS / IONISATION SHOCK

The nuclear spin lattice relaxation time $T_{1\mathrm{n}}$ is known to be exceptionally long [1], which makes nuclear relaxation processes entirely irrelevant within our measurements timescales (and beyond). However, during our experiments, we have observed that the ^{123}Sb nucleus undergoes frequent random spin flips. These nuclear flips must thus originate from the readout process which, relies on the electron acting as an ancilla qubit [2].

In a hyperfine coupled donor system, the spin state of the nucleus (observable) can be measured in the $\hat{I}_z$ basis by first mapping its spin configuration onto the electron spin (ancilla) state by inverting the electron state conditional on one of the nuclear spin projections, i.e. driving the electron at one of the resonant peaks in Fig. 1f. The electron spin is then read out using spin-dependent tunneling into the SET island [3]. An $|\uparrow\rangle$ electron tunnels out to the SET island and is replaced by a $|\downarrow\rangle$. During the time the donor is ionized as a result of this process, the SET current exhibits a spike that can be detected with high fidelity.

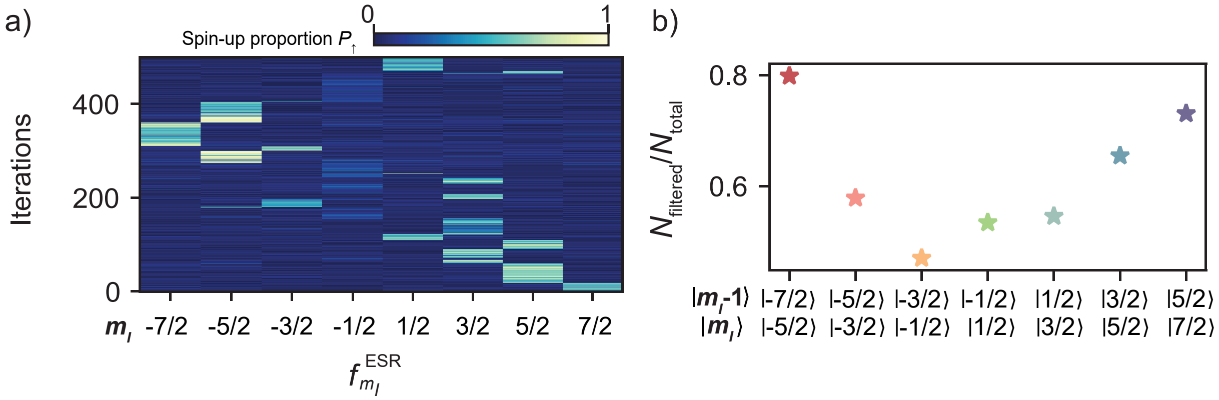

FIG. S1: a) Projective measurements of the nuclear state as a function of time (iterations), measured through electron single-shot readout after applying a microwave stimulus at a frequency $f^{\mathrm{ESR}}_{m_I}$. **b)** Ratio between filtered N_{filtered} and total N_{total} points (see main text for definition), collected from various nuclear ^{123}Sb measurements in this work.

If this is a quantum non demolition (QND) [4] process, the nucleus should remain in the projected state after the ionization event, allowing for repeated (n) measurements to improve the readout accuracy. The condition for QND readout is that the Hamiltonian of the observable $\gamma_{\mathrm{n}} B_0 \hat{I}_z$ commutes with the interaction Hamiltonian with the ancilla $\hat{H}_{\mathrm{in}} = A\hat{\boldsymbol{S}} \cdot \hat{\boldsymbol{I}}$, i.e. $[\hat{H}_{\mathrm{int}}, \hat{I}_z] = 0$. This is true if the interaction Hamiltonian is only given by the secular component of the hyperfine interaction $\hat{H}_{\mathrm{in}} = A\hat{S}_z\hat{I}_z$. However, an accurate description of single-shot readout in a hyperfine-coupled system, requires accounting for the terms in the Hamiltonian that do not commute with $\hat{I}_z$ [2]. These arise from both the isotropic [5] and anisotropic [6] parts of the hyperfine interaction that precede the operators $\hat{S}_\alpha, \hat{I}_\beta$ with $\alpha \neq z$ and $\alpha, \beta \neq z, z$. Moreover, in the case of ^{123}Sb , the removal of the electron could cause a change in the electric field gradient $\mathcal{V}_{\alpha\beta}$ [7], thus modifying the eigenstates between the ionized/neutral atom. These changes in eigenbasis result in a finite probability of flipping the nucleus during the readout process. The phenomenon above is termed *ionization shock*, and in the extreme case where the hyperfine/quadrupolar interaction are highly anisotropic/highly dependent on the charge state, the nuclear states are randomized after each ionization event [6].

Previous work on ion-implanted ^{31}P donors showed that the rate of ionisation shock, i.e. the probability of the nuclear spin flipping as a result of a change in charge state of the donor, can be as low as 10^{-7} [5, 8]. However, much faster nuclear flipping rates have been observed in STM-fabricated donor clusters [6] (with only hyperfine interaction) and for a ^{123}Sb donor [9] (with both hyperfine and quadrupolar interaction).

Figure S1 summarizes some of the observations made on ^{123}Sb in the present device. A first experiment tracks the nuclear spin state after a random initialization of the nucleus. After loading an electron $|\downarrow\rangle$ onto the donor, we apply an adiabatic ESR inversion pulse followed by an electron readout. We repeat this sequence, toggling the frequency of the microwave source between the eight possible ESR resonance frequencies $f^{\mathrm{ESR}}_{m_I}$. A high electron spin-up proportion $P_\uparrow$ after the adiabatic inversion flags the nuclear spin state where the donor is found. The results of these experiments are presented in Fig. S1 a, where each iteration represents the population of the eight possible nuclear spin configurations. We observe regular flips between spin configurations, visible from the meandering high spin-up proportion as a

function of repetitions. A crude estimate of the nuclear spin flip probability *per shot* can be obtained from Fig. S1 a as follows. Each of the 8 data points (one for each nuclear spin orientation) at each iteration is obtained by repeating the nuclear readout process 30 times (shots). However, this does not cause 30×8 ionisation events, because only $|\uparrow\rangle$ electrons leave the donor, plus some 'dark counts' ($|\downarrow\rangle$ electrons escaping the donors accidentally), so we estimate ≈ 50 ionisation events for every iteration. The data shows that the nuclear spin typically flips after $\approx 20 - 50$ iterations. Therefore, the ionisation rate per shot is of order 10^{-3}.

An additional benchmarking parameter is given in Fig. S1 b. Here, we post-analyze the experiments that include nuclear readout (nuclear Rabi oscillations, spectra, Ramsey sequences, etc.) to extract statistics on the nuclear spin flips. Using the outcome of a measurement that involves reading out the nuclear spin [5], we can calculate the fraction of successfully finding the nuclear state in the subspace under investigation (which defines N_{filtered}), after one measurement sequence which typically includes 200-300 electron shots (which defines N_{total}) before the state is reinitialized. Note that this number also captures errors associated with the initialization sequence. We can thus define a probability of 'success' as $N_{\mathrm{filtered}}/N_{\mathrm{total}}$, i.e. the probability that, after performing an experiment intended to measure a property of the system while the nuclear spin is in the m_I projection, we find the nuclear spin still in m_I at the end of the experiment. Unsuccessful experiments are subsequently filtered out of the averages. S1 b Shows that the probability of success is between 0.4 and 0.8, depending on the nuclear state.

SUPPLEMENTARY NOTE 2: DEVICE FABRICATION AND OPERATION

This qudit device was fabricated on a natural silicon wafer with a 900 nm thick epitaxial layer of isotopically enriched ^{28}Si with 800 ppm residual concentration of ^{29}Si. We use electron beam lithography to pattern metallic aluminum structures on top of a thin (≈ 8 nm) SiO_2 oxide layer, to control and readout the donor spins.

The device integrates a broadband microwave antenna [10], which is an on-chip 50 Ω matched coplanar waveguide, terminated by a short circuit. The presence of stray electric fields in the GHz range allows us to use the waveguide to deliver oscillating electric fields.

The device is wire-bonded to a high-frequency printed circuit board mounted within a copper enclosure, which is then bolted to a box where it sits in the air gap of a Halbach array of permanent magnets [11]. The assembly is then anchored to the mixing chamber plate of a Bluefors BF-LD400 dilution refrigerator, where it gets cooled down to $\lessapprox$20 mK. The static magnetic field B_0 ($\approx$1 T) produced by the permanent magnets is applied along the short-circuit termination of the magnetic (ESR, NMR) antenna and parallel to the [110] plane of the Si substrate).

We use flexible copper cables to connect the enclosure to a filter box, attached to the mixing chamber plate which contains two types of low pass filters: second-order low-pass RC filters with a 20 Hz cut-off frequency used for the gates that provide a constant voltage bias to the device (TG, RB, LB, PL), and seventh-order low-pass filters with 80 MHz cut-off frequency, connected to the gates that we use for pulsing (typically donor gates DG and SR) or to measure conductance in the SET (source S and drain D). The gate layout can be found in Fig. S2. Above the filter boxes, the DC lines consist of Constantan looms, which we thermalize by wrapping them around copper rods at various temperature stages. For the fast lines, we use coaxial cables with a graphite coating on the dielectric to minimize the triboelectric effect caused by mechanical vibrations from the pulse tube [12].

For the high-frequency lines, like the on-chip coplanar waveguides, we use silver-plated copper-nickel coaxial cables, with 2.92 mm coaxial connectors, and add a 10 dB attenuator at 4K to thermalize the line. The gates are DC-biased using battery-powered and opto-isolated SRS SIM 928 voltage sources. To increase the voltage resolution we use homemade resistive voltage dividers, with a division of 1:8. The AC signals are generated by arbitrary waveform generators (Keysight M3300A and M3202A), and combined with the DC signals using impedance-matched combiners with a voltage division 1:2.5.

The microwave signals needed for ESR and EDSR are generated by a Keysight E8267D microwave vector source (100 kHz-44 GHz), which we IQ-modulate using the channels from a Keysight 81180A AWG. The NER and NMR control signals are synthesized directly by Keysight M3300A and M3202A AWG cards, which provide bandwidth of up to 500 MS/s and 1 GS/s, respectively. To combine the signals at radio and microwave frequencies to be delivered to the microwave antenna, we use a commercially available diplexer Marki Microwave DPX-1721 at room temperature.

The current from the SET is converted to a voltage using a Femto DLPCA-200 transimpedance amplifier. Typical currents are on the order of 1 nA, thus we use an amplification of 10^7 V/A, to which corresponds an amplifier bandwidth of 50- kHz. The signal is further amplified by an SRS SIM910 JFET amplifier, where we use 100 V/V gain, and filtered by an SRS SIM965 analog 50 kHz low-pass Bessel filter. The converted signal is recorded using the digitizer in the the Keysight M3300A.

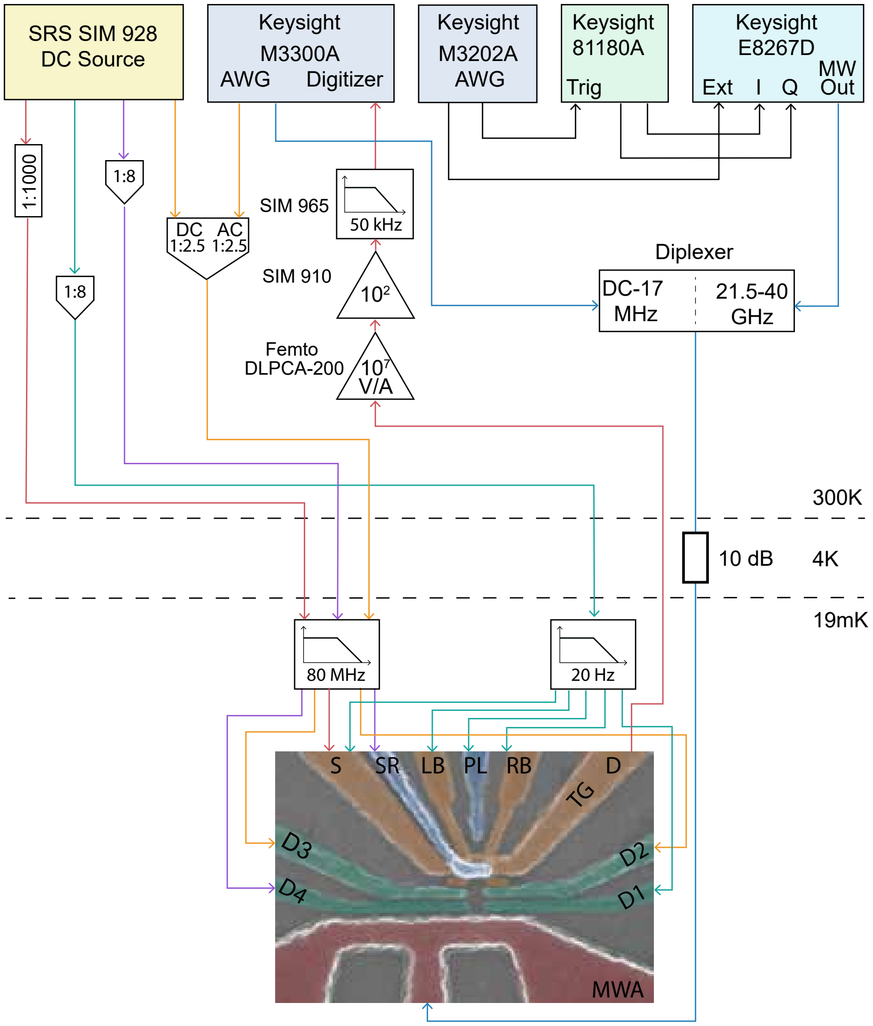

FIG. S2: Schematics the cryogenic setup used to operate the device in this work. The green lines correspond to DC lines (20 Hz cutoff frequency), purple and orange to AC lines (80 MHz cutoff frequency), and the blue line (no filtering) corresponds to the high-frequency line connected to the antenna. All the elements of the diagram, as well as the thermal connections of the cabling, are explained in the main text.

The last step is to interface our instruments at the software level, which is done using a Python-based in-house software called SilQ [13], which uses the Python-based QCoDeS data acquisition framework [14].

SUPPLEMENTARY NOTE 3: NUCLEAR STATE PREPARATION FOR ^{123}SB

By leveraging the flip-flop drive, we can initialize the atom in an arbitrary nuclear subspace without relying on NMR pulses. This is achieved by concatenating sequences that include adiabatic flip-flop and ESR drive, as shown in Fig. S3. This technique enables high-fidelity nuclear state preparation with a compact instrument footprint, and was previously used by Asaad et al.[9].

Using flip-flop initialization requires uploading only two different waveforms (Fig. S3 a and Fig. S3 b) to the AWG, depending on whether we want to increase or decrease the nuclear spin projection.
To increase the nuclear spin number ($|m_I - 1\rangle \rightarrow |m_I\rangle$), we use the sequence presented in Fig.S3 a. We first load an electron $|{\downarrow}\rangle$ from the SET reservoir, and apply two adiabatic ESR pulses which flip the state of the electron un-

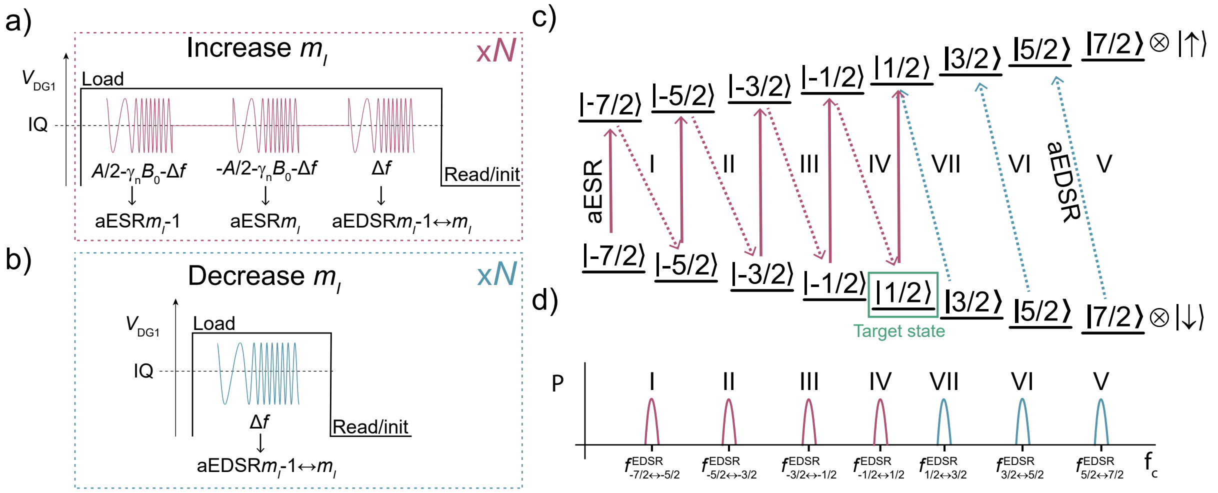

FIG. S3: **a)** Pulse sequence sent to the IQ inputs of the microwave source, used to increase the nuclear spin number $|m_I - 1\rangle \rightarrow |m_I\rangle$, consisting of two adiabatic ESR pulses and one adiabatic EDSR pulse. **b)** Pulse sequence sent to the IQ inputs of the microwave source, used to decrease the nuclear spin number $|m_I\rangle \rightarrow |m_I - 1\rangle$, consisting of one adiabatic EDSR pulse. The frequencies for the pulses in **a)** and **b)** can be generalized by writing them in terms of the value of the hyperfine A, the Zeeman energy $\gamma_n B_0$, and the frequency modulation depth of the adiabatic pulse Δf (see text for details). **c)** Schematics of the transitions involved in the initialization sequence, with the order of the operations indicated with roman numbers. **d)** Depiction of the frequency for the microwave local oscillator, which is set at the EDSR resonance frequency $f^{\mathrm{EDSR}}_{m_I-1\leftrightarrow m_I}$ of each subspace.

conditionally of the state of the nucleus. A subsequent adiabatic EDSR pulse populates the $|m_I\rangle$ subspace only when the electron is found in the $|\uparrow\rangle$ state, therefore increasing the nuclear spin projection . Conversely, to move from $|m_I\rangle \rightarrow |m_I - 1\rangle$, we use the sequence shown in Fig.S3 b consisting of a single adiabatic EDSR pulse, after an electron in the $|\downarrow\rangle$ state has been loaded onto the donor.
This protocol is exemplified in Figures S3 c-d. For a target state $m_I = |1/2\rangle$, we load the pulse sequence in Fig. S3 a to the AWG, and continuously play the waveform while switching the carrier f_c of the microwave source with a software instruction, between the frequencies I,II, III, and IV every $t = t_{\mathrm{ENDOR}}$, where t_{ENDOR} is the duration of the Electron Nuclear Double Resonance (ENDOR) pulse sequence from Fig. S3 a. This should bring any state from the left of $|1/2\rangle$ to the target state. After $N \approx 20$ repetitions of this whole sequence, we upload the pulse sequence in Fig. S3 b and repeat the procedure described above, this time switching the carrier frequency between V,VI,VII. This should bring any initial state that falls on the right-hand side of $|1/2\rangle$ to the target state.

SUPPLEMENTARY NOTE 4: CALCULATION OF QUADRUPOLAR SPLITTING FOR NMR$^0_{\pm1}$

The resonance frequencies for neutral NMR are mathematically obtained by taking the difference in expectation values of the Hamiltonian operator $\hat{H}_{D^0}$ (Eq. 2) between neighbouring nuclear states with a change in nuclear spin projection number $\Delta m = \pm 1$ and $\Delta s = \pm 0$.
The explicit expression for the resonance frequencies is given by

$$\begin{aligned} f^{\mathrm{NMR}^0}_{m_I-1\leftrightarrow m_I} &= \langle\downarrow\uparrow, m_I - 1|\hat{H}_{D^0}|\downarrow\uparrow, m_I - 1\rangle - \langle\downarrow\uparrow, m_I|\hat{H}_{D^0}|\downarrow\uparrow m_I\rangle \\ &= \gamma_n B_0 + \underbrace{\left(m_I - \frac{1}{2}\right) f_q \pm \frac{A}{2}}_{f^1} \pm \underbrace{g_{m_I-1\leftrightarrow m_I}\frac{A^2}{\gamma_e B_0}}_{f^2}, \end{aligned} \tag{S1}$$

where the first term corresponds to the Zeeman splitting at magnetic field B_0 with $\gamma_n = 5.55$ MHz, the second and third terms are the first-order (f^1) contributions of the quadrupolar and isotropic hyperfine interactions, with quadrupole splitting f^0_q and hyperfine constant A, and the fourth term corresponds to the second-order (f^2) contribution of the

hyperfine interaction to the energy splitting, where $g_{m_I-1\leftrightarrow m_I}$ is a coefficient that depends on the nuclear spin projection quantum number.

These coefficients are calculated by considering that each eigenfrequency is corrected to second order by

$$\begin{aligned} f^2_{s,m_I} &= \sum_{m_s',m_I'\neq m_s,m_I} \frac{|\langle m_s', m_I'|\hat{H}_\mathrm{A}|m_s,m_I\rangle|^2}{f^0_{m_s,m_I}-f^0_{m_s',m_I'}}, \\ &= \frac{A^2}{4}\sum_{m_s',m_I'\neq m_s,m_I} \frac{|\langle m_s', m_I'|\left(\hat{S}_+\hat{I}_- + \hat{S}_-\hat{I}_+\right)|m_s,m_I\rangle|^2}{f^0_{m_s,m_I}-f^0_{m_s',m_I'}} \\ &= \frac{A^2}{4}\sum_{m_s',m_I'\neq m_s,m_I} \frac{\left(I\left(I+1\right)-m_I'm_I\right)\left(\delta_{m_s',m_s+1}\delta_{m_I'+1,m_I}+\delta_{m_s'+1,m_s}\delta_{m_I',m_I+1}\right)}{f^0_{m_s,m_I}-f^0_{m_s',m_I'}}, \end{aligned} \tag{S2}$$

where $\hat{H}_A = A\hat{\boldsymbol{S}}\cdot\hat{\boldsymbol{I}}$ is the hyperfine interaction Hamiltonian, which represents the perturbation to the Zeeman eigenbasis, and where $f^0_{m_s,m_I} = \langle m_s,m_I|\,H_\mathrm{Z}\,|m_s,m_I\rangle$ are the Zeeman eigenenergies (in units of Hz). The subscripts $\pm$ assigned to the spin operators $\hat{S}$ and $\hat{I}$ signify whether they represent creation or annihilation operators.

As expected, the Kronecker delta conditions δ in Eq. S2 reveal that corrections from the hyperfine interaction to second order only arise from the associated antiparallel spin states, which are the ones that $\hat{H}_A$ couples. The nuclear-dependent coefficients $g_{m_I-1\leftrightarrow m_I}$ from Eq. S1 are thus calculated using

$$g_{m_I-1\leftrightarrow m_I} = (f^2_{\pm1/2,m_I-1} - f^2_{\pm1/2,m_I})\frac{\gamma_\mathrm{e}B_0}{A^2} \tag{S3}$$

Since the spectrum presented in Fig. 1e corresponds to the subspace where the electron is in the $|{\downarrow}\rangle$ state, our results consider the case where $m_s = -1/2$. This yields $g_{m_I-1\leftrightarrow m_I}$ = [1.75, 1.25, 0.75, 0.25, -0.25,-0.75, -1.25] for $m_I \in \{-5/2,...,7/2\}$.

To obtain the quadrupolar splitting from the experimental $\mathrm{NMR}^0_{\pm1}$ resonance frequencies, we first need to estimate the value of the hyperfine interaction strength A. Conveniently, we can do this without considering the quadrupolar splitting f_q, as its contribution to the transition frequencies is symmetric with respect to the nuclear spin number. This means that we can find an expression that only depends on the unknown value A by adding the transition frequencies with same absolute value of the nuclear spin projection,

$$f^{\mathrm{NMR}^0}_{m_I-1\leftrightarrow m_I} + f^{\mathrm{NMR}^0}_{m_{-I}\leftrightarrow m_{-I}+1} = 2\gamma_\mathrm{n}B_0 + A + \frac{A^2}{2\gamma_\mathrm{e}B_0}, \tag{S4}$$

where $B_0 = 999.5(5)$ mT was obtained from the ionised NMR spectrum in Fig. 1d and where $f^{\mathrm{NMR}^0}_{mI-1\leftrightarrow m_I}$ correspond to the experimental resonance frequencies for the neutral nucleus in Fig. 1e. The quadratic equation S4 can be solved exactly and the value of the hyperfine is calculated for the case where $m_I \in \{-\frac{1}{2},-\frac{3}{2},-\frac{5}{2}\}$. Using these three solutions we obtain an average value of the hyperfine interaction of $A = 96.584(2)$ MHz. By substituying this quantity in Eq. S1, we calculate the quadrupolar splitting from the distance between transitions with $\Delta m = 1$, resulting in $f_q = -52.5(5)$ kHz for the neutral atom.

SUPPLEMENTARY NOTE 5: CALCULATION OF HYPERFINE INTERACTION FROM ESR SPECTRUM

To obtain the resonance frequencies for the electron spin, we mathematically calculate the difference in the expectation values of the Hamiltonian operator $\hat{H}_{D^0}$ (as defined in Eq. 2) when the nuclear spin projection number is fixed at $\Delta m = 0$ and there is a change in the electron spin projection number, $\Delta s = 1$:

$$f^{\mathrm{ESR}}_{m_I} = \langle\uparrow m_I|\hat{H}_{\mathrm{D}^0}|\uparrow m_I\rangle - \langle\downarrow m_I|\hat{H}_{\mathrm{D}^0}|\downarrow m_I\rangle = \gamma_\mathrm{e}B_0 + \underbrace{m_IA}_{f^1} + \underbrace{b_{m_I}\frac{A^2}{\gamma_\mathrm{e}B_0}}_{f^2}, \tag{S5}$$

where we noted the first- (f^1) and second- (f^2) order corrections resulting from the hyperfine interaction. The latter arise from the transversal components of the hyperfine interaction and correct the electron eigenenergies dependent on

the state of the nuclear spin (whereas the quadrupolar interaction term leaves them unaffected).
Using Eq. S2, we obtain that each ESR resonance peak shown in Fig. 1f is corrected to second order by

$$f_{m_I}^{2,\mathrm{ESR}} = f^2_{\frac{1}{2},m_I} - f^2_{-\frac{1}{2},m_I}. \tag{S6}$$

Taking the difference between adjacent nuclear states shows that the distance between ESR features a second order contribution from A that depends on the nuclear spin projection

$$f_{m_I}^{\mathrm{ESR}} - f_{m_I-1}^{\mathrm{ESR}} = A + (f_{m_I}^{2,\mathrm{ESR}} - f_{m_I-1}^{2,\mathrm{ESR}}) = A\left(1 + c_{m_I-1\leftrightarrow m_I}\frac{A}{(\gamma_\mathrm{e}+\gamma_\mathrm{n})\,B_0}\right), \tag{S7}$$

where the values for $c_{m_I-1\leftrightarrow m_I}$ are given in Table. S1

(m_I-1, m_I)	$(-\frac{7}{2},-\frac{5}{2})$	$(-\frac{5}{2},-\frac{3}{2})$	$(-\frac{3}{2},-\frac{1}{2})$	$(-\frac{1}{2},\frac{1}{2})$	$(\frac{1}{2},\frac{3}{2})$	$(\frac{3}{2},\frac{5}{2})$	$(\frac{5}{2},\frac{7}{2})$
$c_{m_I-1\leftrightarrow m_I}$	3	2	1	0	-1	-2	-3

TABLE S1: Coefficients for second order corrections to the hyperfine interaction between pairs of nuclear states

Since $c_{-1/2\leftrightarrow 1/2} = 0$ in this case, the value of A can be obtained by calculating the difference between $f_{1/2}^{\mathrm{ESR}}$ and $f_{-1/2}^{\mathrm{ESR}}$, which yields $A = 95.442(22)\,\mathrm{MHz}$. The disparities observed in the values of A obtained from the NMR and ESR spectra may arise due to varying electrostatic configurations between the experiments.

SUPPLEMENTARY NOTE 6: RABI OSCILLATIONS

In this section we collate the raw data on the Rabi oscillations for the electron and nuclear spin transitions. In these experiments, a single frequency tone modulated by a baseband pulse is applied at each of the frequencies shown in Fig.1 d-i, with a varying pulse duration. By varying the duration of the control pulse, Rabi oscillations are observed (Fig. S3-S5), demonstrating coherent control on each pair of sublevels. Fitting the data in Fig. S3-S5, we extract the values of f_{Rabi} reported in Fig. 2.

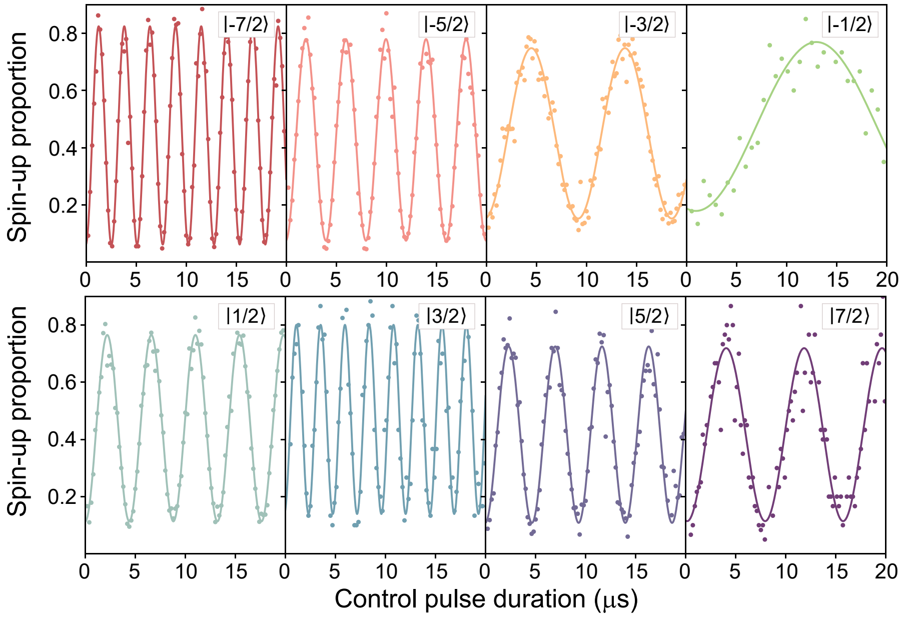

FIG. S4: ESR Rabi oscillations of a ^{123}Sb donor. Electron spin Rabi oscillations on all eight ESR frequencies, where the state of the nuclear spin $|m_I\rangle$ for each panel is indicated in the legend. The ESR pulse amplitude at the source ($V_{\rm MW}^{\rm pp} = 300\,{\rm mV}$) in constant in all measurement. The different Rabi frequencies are attributed to a frequency-dependent transmission of the microwave antenna.

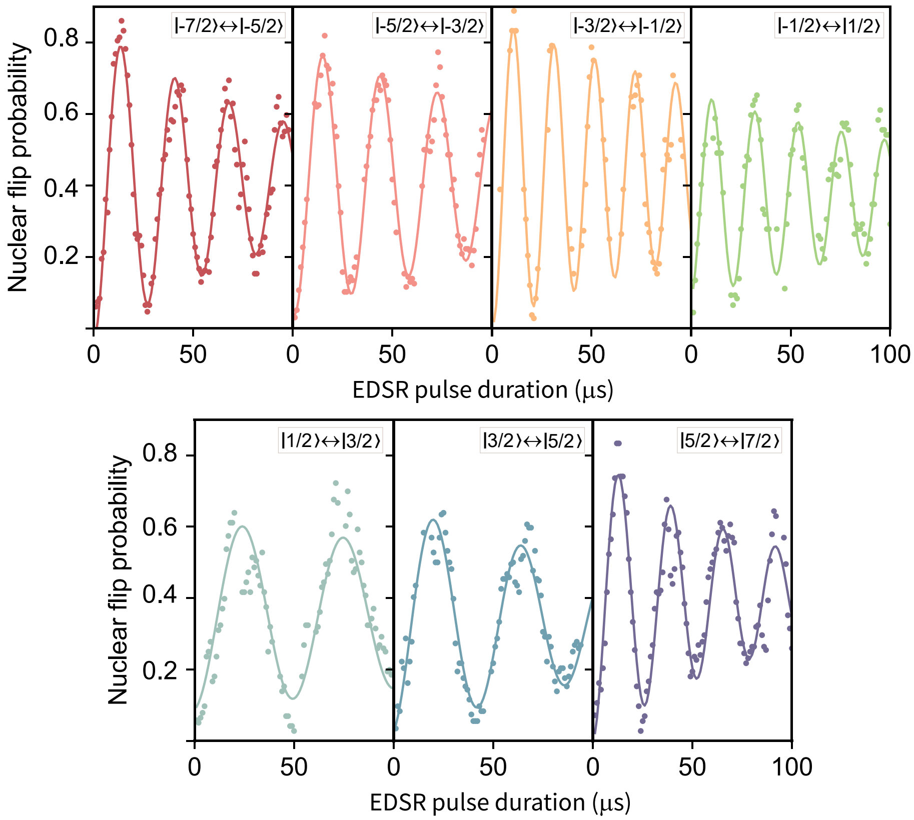

FIG. S5: EDSR Rabi oscillations of the ^{123}Sb donor. Electric drive of the flip-flop transitions using the high-frequency microwave antenna, with a pulse amplitude of $V_{\mathrm{MW}}^{\mathrm{pp}} = 300\,\mathrm{mV}$ at the source. The insets indicate the nuclear subspace. The data points have been smoothened using a Savitzky–Golay filter, with a window length of 3 and a degree 1 polynomial to soothe an oversampling of the curves along the x-axis.

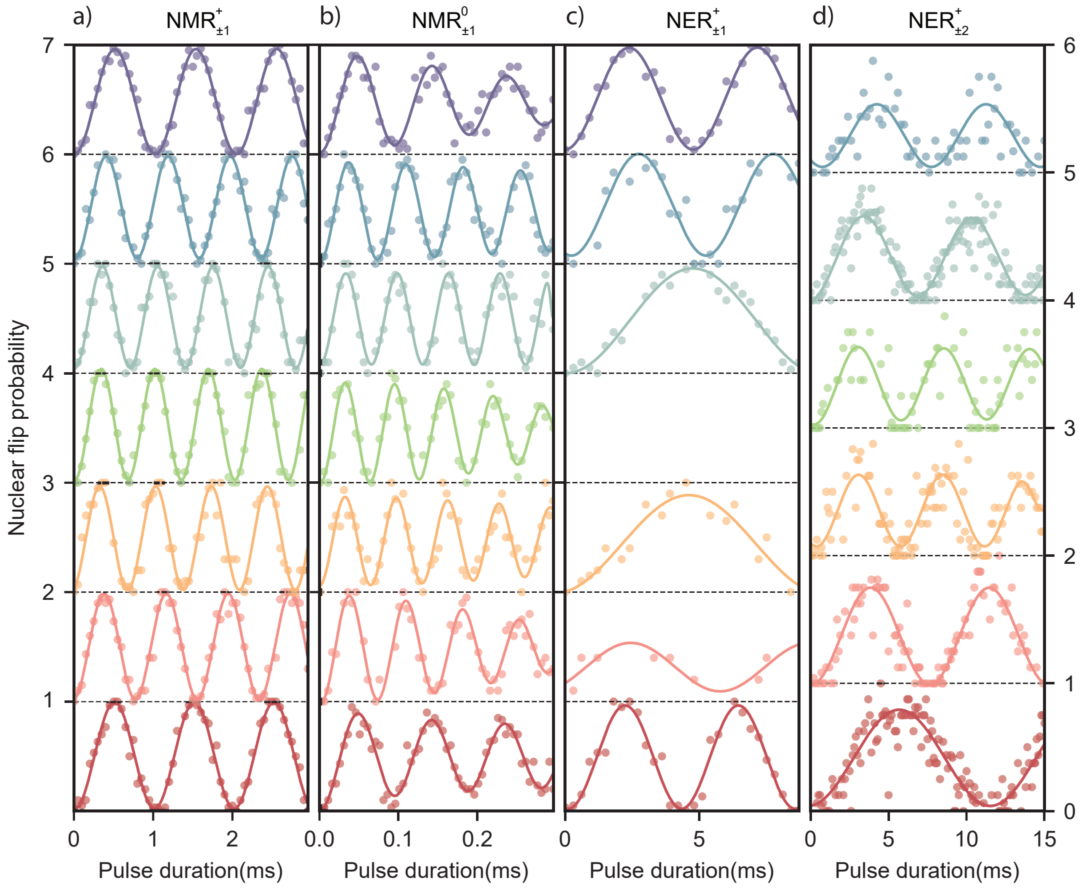

FIG. S6: Coherent nuclear spin drive of an ^{123}**Sb donor. a), b)** Magnetically driven Rabi oscillations on the **a)** ionised (NMR$^+_{\pm1}$), and **b)** neutral (NMR$^0_{\pm1}$) nucleus in the $|{\downarrow}\rangle$ electron spin configuration. **c), d)** Coherent electric drive on the ionised nuclear spin for **c)** $\Delta m_I = 1$ (NER$^+_{\pm1}$), where the fourth row is left blank as driving $|-1/2\rangle \leftrightarrow |1/2\rangle$ is not allowed with NER drive (See Tab. 1), and **d)** $\Delta m_I = 2$ (NER$^+_{\pm2}$).

SUPPLEMENTARY NOTE 7: CALCULATION OF THE STARK EFFECT

To calculate the Stark shift of the spectral lines, we examine the change in resonance frequencies as a function of bias voltage, presented experimentally in Fig. 3a,b of the main text. These can be derived analytically by calculating the derivative of the electron ($f^{\rm ESR}_{m_I}$) and nuclear ($f^{\rm NMR^0}_{m_I-1\leftrightarrow m_I}$, $f^{\rm NMR^+}_{m_I-1\leftrightarrow m_I}$) resonance frequencies (Table. 1 of the main text) as a function of electric field (which in our devices is represented by a change in voltage V).

A. Electron spin

Starting with the electron we have

$$f^{\rm ESR}_{m_I} = \gamma_{\rm e}(E)B_0 + m_I A(E) + b_{m_I}\frac{A(E)^2}{B_0\gamma_{\rm e}(E)}, \tag{S8}$$

where we have indicated the terms that are sensitive to static electric fields. The nuclear-state dependent coefficients b_{m_I} are obtained from Eq. S7. Taking the derivative yields

$$\frac{\partial f^{\rm ESR}_{m_I}}{\partial V} = \frac{\partial \gamma_{\rm e} B_0}{\partial V} + m_I\frac{\partial A}{\partial V} + 2b_{m_I}\frac{A}{B_0\gamma_{\rm e}}\frac{\partial A}{\partial V}. \tag{S9}$$

We note that $\gamma_{\rm e}$ in the denominator of $b_{m_I}A^2/B_0\gamma_{\rm e}$ should in principle be included in the derivative. However, we can safely ignore its contribution as it scales as $A^2/\gamma_{\rm e}^2$. The symmetry of Eq. S9 with respect to the nuclear spin number allows us to extract the slopes $\partial A/\partial V$ by substracting the expressions with opposite nuclear spin number

$$\frac{\partial f^{\rm ESR}_{m_I}}{\partial V} - \frac{\partial f^{\rm ESR}_{m_{-I}}}{\partial V} = 2m_I\frac{\partial A}{\partial V}. \tag{S10}$$

After this calculation, we can obtain the g-factor (gyromagnetic ratio) Stark effect, if we instead sum the derivatives with opposite nuclear spin projection

$$\frac{\partial f^{\rm ESR}_{m_I}}{\partial V} + \frac{\partial f^{\rm ESR}_{m_{-I}}}{\partial V} = 2\frac{\partial \gamma_{\rm e} B_0}{\partial V} + 4b_{m_I}\frac{A}{B_0\gamma_{\rm e}}\frac{\partial A}{\partial V}. \tag{S11}$$

We extract the experimental slopes $\partial f^{\rm ESR}_{m_I}/\partial V$ by performing linear fits to the data in Fig. 3 a and use Eq. S10 and Eq. S11 to obtain the Stark effect on A and $\gamma_{\rm e}$ for each subspace $|m_I\rangle$. We calculate an average value of $\partial A/\partial V = 9.8(4)$ MHz/V and $\partial\gamma_{\rm e}B_0/\partial V = -1.4(6)$ MHz/V, where the errobars represent the standard error.
Using the extracted slopes, we numerically obtain the Stark effect on the ESR resonance lines by calculating the eigenenergies using the Hamiltonian from Eq. 2, as a function of voltage amplitude. The results are presented with the solid lines in Fig. 3b of the main text.

Note that we have excluded the data for $m_I = 1/2$ in the analysis since we observe a large deviation from the expected trend. We attribute this deviation from the expected trend to a sudden jump in the ESR frequency during the experiment, caused by a charge rearrangement, or a hyperfine-coupled ^{29}Si, which erroneously attributes a change of gate voltage $\Delta V^{\rm DG1}_{\rm DC}$ to a change in resonance frequency $\Delta f_{\rm ESR}$.

B. Nuclear spin (neutral donor)

In the neutral antimony atom (^{123}Sb^{0}), both the hyperfine coupling and the quadrupolar interaction are sensitive to changes in the local electric field [9, 15–17]. The explicit formula for the resonance frequencies of the neutral ^{123}Sb atom, denoting the electric field dependency on the Hamiltonian parameters, is given by

$$f^{\rm NMR^0}_{m_I-1\leftrightarrow m_I} = \gamma_{\rm n}B_0 + \left(m_I - \frac{1}{2}\right) f_{\rm q}(E) \pm \frac{A(E)}{2} \pm g_{m_I-1\leftrightarrow m_I}\frac{A(E)^2}{\gamma_{\rm e}B_0}, \tag{S12}$$

where $f_\mathrm{q}(E)$ is the E-field dependent value for the quadrupolar splitting, and $g_{m_I-1\leftrightarrow m_I}$ are the nuclear spin-dependent coefficients that scale the second order corrections from A, and are derived in Supplementary Information S4. Calculating the derivative, we find

$$\frac{\partial f^{\mathrm{NMR}^0}_{m_I-1\leftrightarrow m_I}}{\partial V} = \left(m_I - \frac{1}{2}\right)\frac{f_\mathrm{q}}{\partial V} \pm \frac{1}{2}\frac{\partial A}{\partial V} \pm g_{m_I-1\leftrightarrow m_I}\frac{2A}{\gamma_\mathrm{e}B_0}\frac{\partial A}{\partial V}. \quad \text{(S13)}$$

In this derivation, we are also ignoring the electric-field-dependence of γ_e, as we did for the electron in section S7A. We can see from Eq. S13 that a change in the hyperfine coupling results in the same spectral shift for all nuclear spin projections to first order ($\frac{\partial A}{2\partial V}$), contrary to what we saw for ESR. However, different slopes arise as we evaluate the second order contributions from the hyperfine interaction, as well as the changes in the quadrupolar splitting.
Using the same approach as we did for ESR (Eq. S10 and Eq. S11) we now obtain the following relations

$$\begin{aligned}\frac{\partial f^{\mathrm{NMR}^0}_{m_I-1\leftrightarrow m_I}}{\partial V} - \frac{\partial f^{\mathrm{NMR}^0}_{m_{-I}\leftrightarrow m_{-I}+1}}{\partial V} =& 2(m_I - \frac{1}{2})\frac{\partial f_q}{\partial V} + \\ &+ (g_{m_I-1\leftrightarrow m_I} - g_{m_{-I}\leftrightarrow m_{-1}+1})\frac{2A}{\gamma_\mathrm{e}B_0}\frac{\partial A}{\partial V},\end{aligned} \quad \text{(S14)}$$

and

$$\frac{\partial f^{\mathrm{NMR}^0}_{m_I-1\leftrightarrow m_I}}{\partial V} + \frac{\partial f^{\mathrm{NMR}^0}_{m_{-I}\leftrightarrow m_{-I}+1}}{\partial V} = \frac{\partial A}{\partial V}\left(1 + (g_{m_I-1\leftrightarrow m_I} + g_{m_{-I}\leftrightarrow m_{-I}+1})\frac{2A}{\gamma_\mathrm{e}B_0}\right), \quad \text{(S15)}$$

where $m_I \in \{-\frac{1}{2}, -\frac{3}{2}, -\frac{5}{2}\}$. After performing linear fits to the data in Fig. 3b, we use Eq. S15 to extract $\partial A/\partial V$ for each nuclear subspace, using $A = 96.584(2)$ MHz, and $B_0 = 999.5(5)$ mT from the ionized ^{123}Sb donor spectrum. We obtain an average value of $\partial A/\partial V = 11.6(5)\mathrm{MHz/V}$, where the error bars are expressed as the standard error. This result can be used to retrieve the contribution from the quadrupole Stark effect using Eq. S14. In this case, it is important that we consider the second order terms from A, as they might be on the order of the quadrupolar shifts. We obtain an average value of $\partial f_q/\partial V \approx -300(56)\,\mathrm{kHzV^{-1}}$. As we did for the electron, we use these results to numerically calculate the Stark effect on the nuclear resonance frequencies, by solving the Hamiltonian in Eq. 2 as a function of voltage gate $V_\mathrm{DC}^\mathrm{DG1}$ (see main text). The calculated values are presented as solid lines in Fig. 3a of the main text.

C. Nuclear spin (ionised donor)

The ionised donor nucleus is only sensitive to electric fields via the linear quadrupole Stark effect (LQSE). Here we present the data from which the LSQE on the ionised nucleus, $\partial f_\mathrm{q}^+/\partial V = -2.07(2)\,\mathrm{kHz/V}$, was extracted. The data in Fig. C is obtained from an NMR spectrum for the ionised donor, taken as a function of the DC bias amplitude $V_\mathrm{DC}^\mathrm{DG1}$. The inset shows a linear fit to the data, where the slope has been scaled by a factor of $(m_I - 1/2) = 3$ for this transition.

SUPPLEMENTARY NOTE 8: QUADRATIC NUCLEAR STARK EFFECT

Understanding the hyperfine shift caused by electric fields requires knowledge of the donor's distance to the interface (depth). For donors in bulk silicon, the hyperfine Stark shift is quadratic in the electric field. [18]. However, as the donor depth decreases, the change in hyperfine coupling becomes linearly dependent on the electric field. The total hyperfine shift can be expressed as follows:

$$\Delta A(\boldsymbol{E}) = A(0)\left(\eta_1 E + \eta_2 E^2\right), \quad \text{(S16)}$$

where $A(0) = |\psi(0, r_0)|^2$ represents the hyperfine value in the absence of an electric field, and $\eta_1(\mathrm{\mu m/V})$ and $\eta_2(\mathrm{\mu m^2/V^2})$ are parameters that define the strength of the linear and quadratic Stark effects, respectively.

In order to characterize the quadratic contributions of electric fields $\eta_2 E^2$ from Eq. S16, we choose an echo refocusing pulse scheme like the one outlined in Ref. [19]. We concentrate our analysis on the subspace $|{\downarrow}, 7/2\rangle \leftrightarrow |{\downarrow}, 5/2\rangle$,

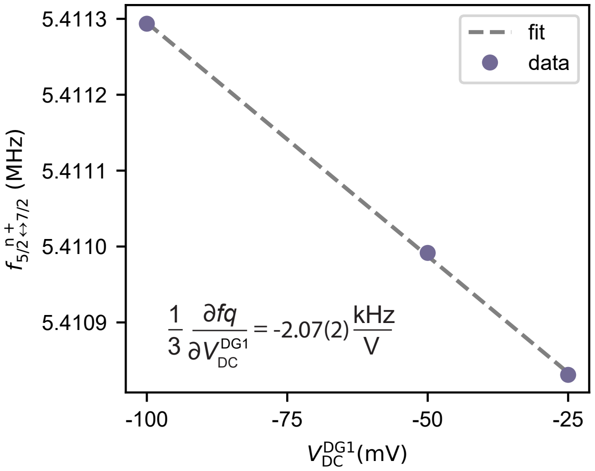

FIG. S7: LQSE $\Delta m = 1$ for the ionised ^{123}Sb donor. Linear quadrupolar Stark effect measured for the transition $|5/2\rangle \leftrightarrow |7/2\rangle$ in the ionised nucleus.

and use magnetic control (NMR) to drive the nuclear spin. The modified Hahn echo works as follows: We start by bringing the nuclear spin to the xy-plane with a $X_{\pi/2}$ pulse, where we let the spin precess for a time τ. During this time, we choose to (i) wait (Fig. S8 a), (ii) apply a 'unipolar' voltage pulse with amplitude V_{DC} (Fig. S8 b) or (iii) apply a 'bipolar' voltage pulse with amplitude $\pm V_{\mathrm{DC}}$ (Fig. S8 c) to one of the donor gates. We then invert the spin with a refocusing X_π pulse, let it precess for another time τ and project it back to the $z-$axis with a final $X_{\pi/2}$ pulse.
The Hahn echo sequence cancels out dephasing during the free precession time τ if the phase accumulated is constant during both τ periods. However, the bipolar and unipolar pulse make the spin accumulate a phase $\theta_{m_I-1\leftrightarrow m_I}(\tau)$ during the first interval τ:

$$\theta_{m_I-1\leftrightarrow m_I}(\tau) = 2\pi\Delta f^{\mathrm{NMR}^0}_{m_I-1\leftrightarrow m_I}\tau, \tag{S17}$$

where

$$\Delta f^{\mathrm{NMR}^0}_{m_I-1\leftrightarrow m_I} = \frac{\partial f^{\mathrm{NMR}^0}_{m_I-1\leftrightarrow m_I}}{\partial V}\Delta V, \tag{S18}$$

$$= \frac{\partial f^{\mathrm{NMR}^0}_{m_I-1\leftrightarrow m_I}}{\partial V}V_{\mathrm{DC}}, \tag{S19}$$

is the change in resonance frequency caused by the pulse with an amplitude $\Delta V = V_{\mathrm{DC}}$. In both cases, the phase accumulated during both free precession periods is different and the echo pulse X_π does not refocus the spin. From Eq. S16 and Eq. S17 we see that in the absence of a quadratic contribution from the electric field, the bipolar pulse compensates the accumulated phase during the free precession time by accumulating a positive phase $\theta_{m_I-1\leftrightarrow m_I}(\tau/2)$, and a negative phase $-\theta_{m_I-1\leftrightarrow m_I}(\tau/2)$ of equal magnitude. In the presence of a quadratic contribution, the sign of $\theta_{m_I-1\leftrightarrow m_I}(\tau/2)$ does not change and thus gives a net accumulated phase of $2|\theta_{m_I-1\leftrightarrow m_I}(\tau/2)|$. For a unipolar pulse, the spin accumulates a phase during the first time τ from both the linear $\eta_1 E$ and quadratic $\eta_2 E^2$ contributions of the electric field.

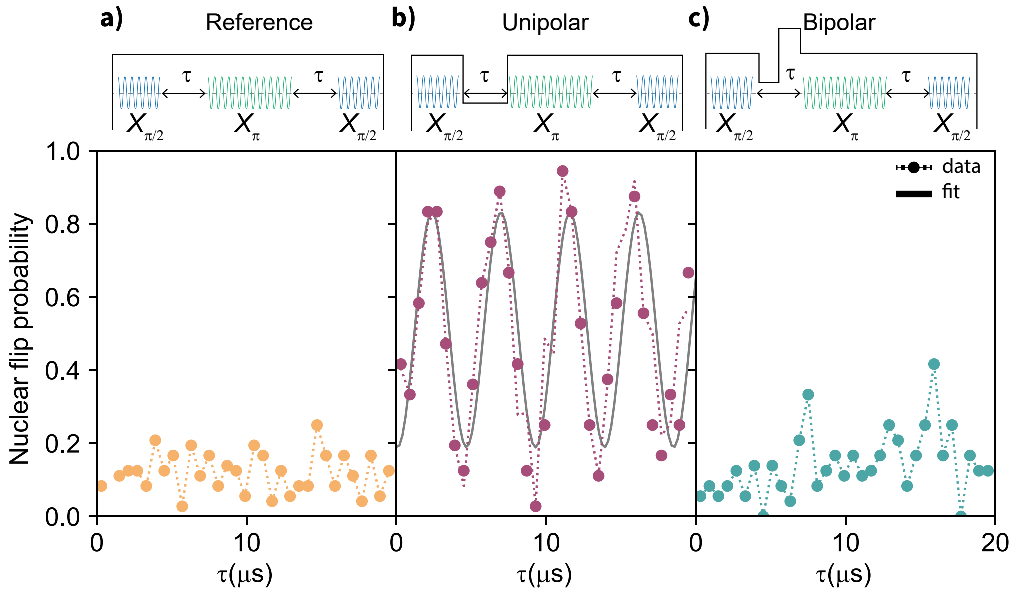

FIG. S8: Linear and quadratic hyperfine Stark effect on a neutral ^{123}Sb^{0} donor. a) Hahn echo experiment on the neutral nucleus, without unipolar/bipolar pulses, used as a reference. As expected, the absence of electric pulses during the free precession causes no phase accumulation. **b)** Hahn echo experiment with a unipolar voltage pulse of amplitude $V_{\mathrm{DC}} = 40\,\mathrm{mV}$ during the first free precession time τ. An accumulated phase as a function of τ is resolved in the oscillatory return probability. The unipolar pulse causes the spin to accumulate a phase given by Eq. S17. **c)** Hahn echo experiment with a bipolar pulse with amplitude $\pm V_{\mathrm{DC}} = 40\,\mathrm{mV}$ and duration $\tau/2$. The lack of oscillations indicates that the quadratic contribution is small and its effect cannot be resolved for $\tau < 20\mu$s.

The data for these experiments is presented in Fig. S8 a-c. We confirm that the absence of an electric pulse causes no phase accumulation in the nuclear spin, and the return probability stays flat (Fig. S8 a).
When a unipolar pulse of amplitude $V_{\mathrm{DC}} = 40\,\mathrm{mV}$ is applied, we observe that the return probability oscillates. The oscillation frequency is obtained by fitting the data to a sinusoidal function $P\sin(2\pi f t + \phi) + P_{\mathrm{offset}}$, and using Eq. S17 and Eq. S18 with $\theta_{-7/2\leftrightarrow-5/2} = \pi$ and $\tau = 2.3\,\mu$s we calculate a $\Delta f^{\mathrm{NMR}^0}_{m_I-1\leftrightarrow m_I} = 217(2)\,\mathrm{kHz}$. Using the the estimated parameters for the Stark effect obtained from directly measuring the spectrum as a function of gate voltage (Fig. 4. b), we find $\Delta f^{\mathrm{NMR}^0}_{m_I-1\leftrightarrow m_I} = 235(3)\,\mathrm{kHz}$,showing an excellent agreement between the two methods.
The experiment with the bipolar pulse, shown in Fig. S8 c, displays no visible oscillation within the chosen evolution time, i.e. no detectable quadratic Stark effect. This is consistent with the expected behaviour of a donor close to an interface, and subjected to a strong static electric field.

SUPPLEMENTARY NOTE 9: ELECTRON T_1 TIME

We measure the relaxation time for the electron in the lowest electron-nuclear energy state, free of flip-flop transition. After initializing the nuclear state using the method outlined in Supplementary Section 3, we load an electron in the $|\downarrow\rangle$ state, and invert it to the $|\uparrow\rangle$ state using an adiabatic ESR pulse. While monitoring the spin-up population as a function of t_{wait} (which equals the time between excitation to $|\uparrow\rangle$ and electron readout), we can measure the decay in spin-up fraction, due to the relaxation of the electron spin to the $|\downarrow\rangle$ state. At $B_0 \approx 1T$ we measure $T_{1e} = 2.44(17)\,\mathrm{s}$ (Fig. S9), which is similar to the typical values found in ^{31}P donor electrons near a SiO_2 interface [20].

SUPPLEMENTARY NOTE 10: COHERENCE TIMES

We measured the coherence times for the electron spin of the ^{123}Sb atom, in the nuclear state $m_I = |7/2\rangle$. Figure S10 a shows a Ramsey experiment performed on the electron. By fitting the curve to a Gaussian decaying sinusoid

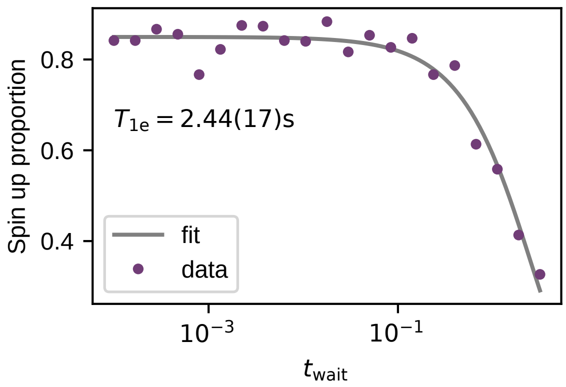

FIG. S9: Electron spin relaxation time T_{1e} Electron spin relaxation for ^{123}Sb measured in the nuclear state $m_I = 7/2$. The data is fitted using an exponentially decaying function $P_0 \exp(-t_{wait}/T_{1e}) + P_{offset}$, revealing a relaxation time of the electron into the $|{\downarrow}\rangle$ state of $T_{1e} = 2.44(17)$ s.

$P \exp(-\tau/T_{2e}^*)^2 \sin(2\pi f\tau + \phi) + P_{offset}$, we obtain $T_{2e}^* = 11.05(64)$ μs. Note that for this Ramsey experiment, we detune the $X_{\pi/2}$ pulse from the resonance frequency $f_{7/2}^{ESR}$, resulting in oscillations the spin-up proportion as a function of τ, known as the Ramsey fringes. Also, note that the duration of the $X_{\pi/2}$ pulse is calibrated to give a maximum return probability (spin-up proportion) for $\tau = 0$ for a $X_{\pi/2}$ pulse on resonance with $f_{7/2}^{ESR}$.

We further characterize the coherence times of the ^{123}Sb electron with a Hahn echo experiment. The decoupling sequence extends the coherence times of the electron, as shown in Fig. S10 b where we measure $T_{2e}^{H} = 510(36)$ μs obtained by fitting the decaying curve to $P_{offset} - P \exp(-\tau/T_{2e}^{H})^{\beta_e^H}$ where $\beta_e^H = 1.67(25)$.

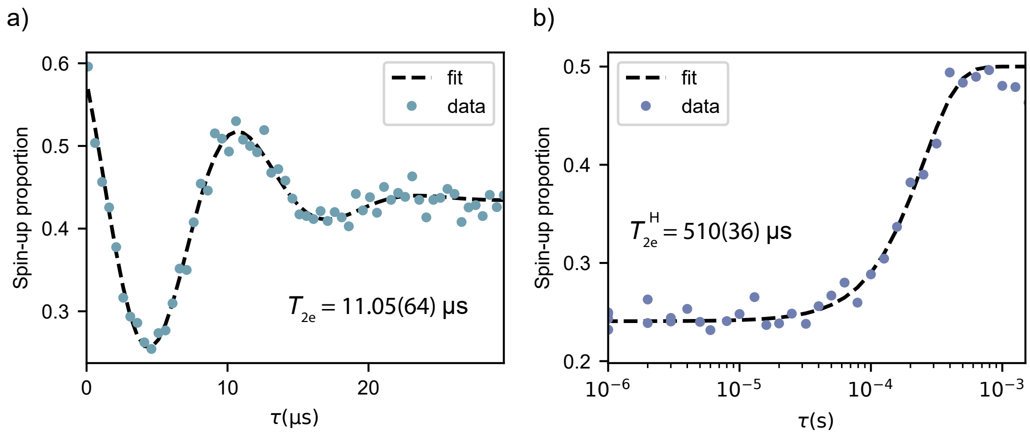

FIG. S10: ^{123}Sb electron coherence times. a) Off-resonant Ramsey experiment ($X_{\pi/2} - \tau - X_{\pi/2}$) on the electron of a ^{123}Sb donor for the transition with $m_I = 7/2$. The mean of 11 repetitions is fitted to a Gaussian decaying sinusoid (see text) and reveals a $T_{2e}^* = 11.06(64)$ µs. **b)** Hahn echo experiment ($X_{\pi/2} - \tau - X_{\pi} - \tau - X_{\pi/2}$) on the electron for the transition $m_I = 7/2$. The decaying curve is fitted with an exponentially decaying function (see main text), and reveals a $T_{2e}^{H} = 510(36)$ µs and a $\beta_e^H = 1.67(25)$.

We next measured the dephasing times on the neutral nucleus using a Hahn echo sequence. The mean of five repetitions (Fig. S11 c) is fitted to an exponential decay $P_{offset} - P \exp(-\tau/T_{2n0}^{H})^{\beta_{n0}^H}$, from which we extract a coherence time $T_{2n0}^{H} = 247(41)$ μs. We note that this value sets a lower bound to the Rabi rates needed to observe coherent drive on the neutral atom. This potentially explains our inability to achieve electrical control over the neutral nucleus in this work, since NER is a slow process, with typical Rabi periods on the order of milliseconds (Fig.S6 c-d).

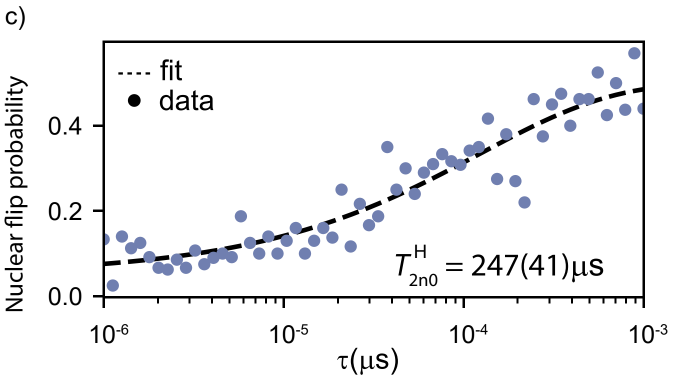

FIG. S11: ^{123}**Sb nuclear coherence times.** Hahn echo experiment on the transition $|5/2\rangle \leftrightarrow |7/2\rangle$. The decay is fitted with an exponentially decaying curve $P_{\mathrm{offset}} - P\exp\left(-\tau/T^{\mathrm{H}}_{2\mathrm{n}0}\right)^{\beta^{\mathrm{H}}_{\mathrm{n}0}}$. The fitting reveals a $T^{\mathrm{H}}_{2\mathrm{n}0} = 247(11)$ µs.

SUPPLEMENTARY NOTE 11: GST EXPERIMENTS

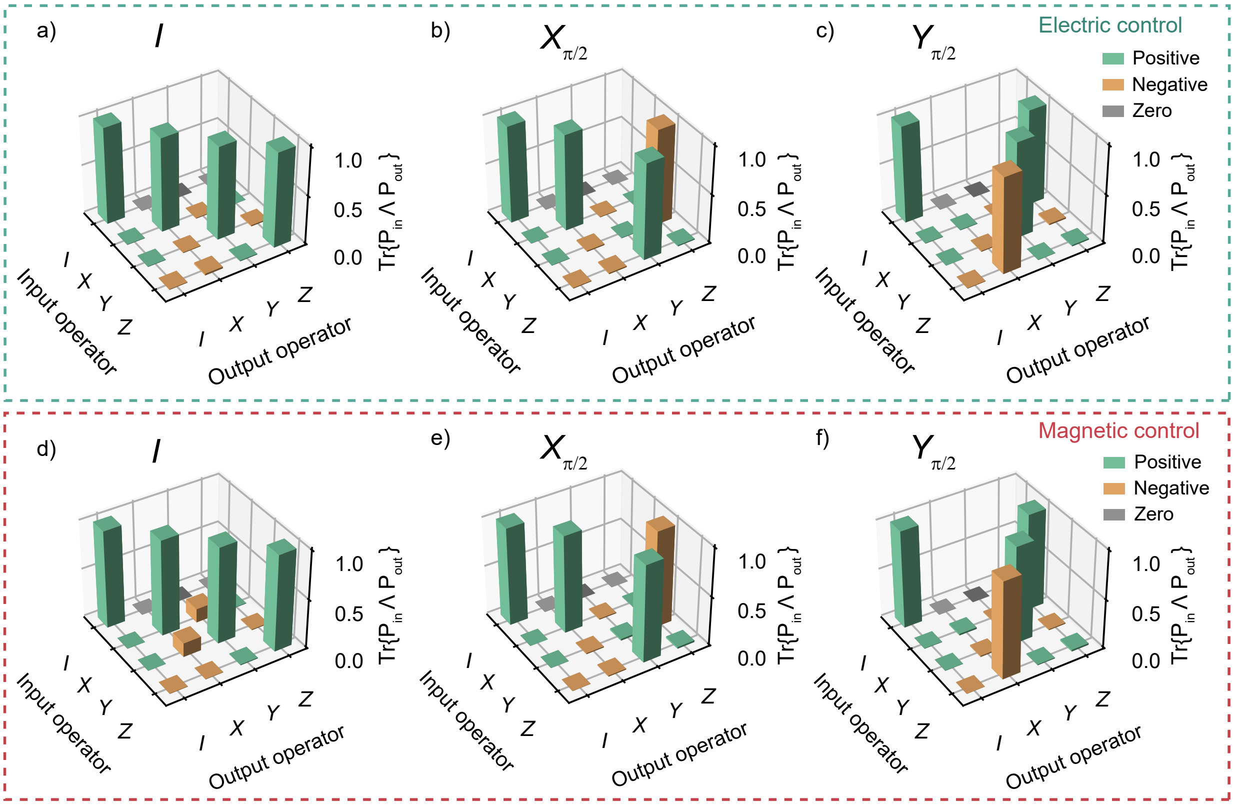

FIG. S12: Process matrices for ionized nuclear 1Q-GST. **a)-c)** Estimated process matrices for the qubit gates a) $\mathbb{I}$, b) $X_{\pi/2}$ and c) $Y_{\pi/2}$, obtained using NER drive. **d)-e)** Estimated process matrices for the qubit gates d) $\mathbb{I}$, e) $X_{\pi/2}$ and f) $Y_{\pi/2}$, obtained using NMR drive. In both cases, a circuit depth of L = 8 was used, corresponding to 448 circuits.

The one-qubit GST experiment aims to investigate the performance of the gates $\mathbb{I}$ (idle gate), $X_{\pi/2}$ (a $\pi/2$ rotation around the x-axis), and $Y_{\pi/2}$ (a $\pi/2$ rotation around the y-axis). In our experiments, we apply NMR/NER on-resonance

pulses to the magnetic antenna/donor gate for the $X_{\pi/2}$ and $Y_{\pi/2}$ gates, while the idle gate $\mathbb{I}$ was an off-resonant NMR/NER pulse detuned by 1 MHz from the resonance frequency.

To create the gate set, we used six fiducial sequences, namely, ($\mathbb{I}$, $X_{\pi/2}$, $Y_{\pi/2}$, $X_{\pi/2}X_{\pi/2}X_{\pi/2}$, $Y_{\pi/2}Y_{\pi/2}Y_{\pi/2}$, $X_{\pi/2}X_{\pi/2}$), where $X_{\pi/2}$ ($Y_{\pi/2}$) are noisy $\pi/2$ rotations around x (y). These fiducials map the qubit density matrix ρ to the six Pauli eigenstates, defining an informationally complete experimental reference frame. We then selected the smallest set of germs ($\mathbb{I}$, $X_{\pi/2}$, $Y_{\pi/2}$, $X_{\pi/2}Y_{\pi/2}$, $X_{\pi/2}X_{\pi/2}Y_{\pi/2}$) that amplified errors and included them in the gate set. Using these sets of fiducials and germs, we created the circuit list using the open-source software pyGSTi [21].

We set the circuit depth to $L = 8$, which limits the maximum number of gates in each circuit to 8 and results in a list of 448 different circuits. We chose $|-5/2\rangle = |0\rangle$ and $|-7/2\rangle = |1\rangle$ as the computational basis. After a measurement, statistics for each circuit are gathered as a binary count of $|0\rangle$ and $|1\rangle$. We use the software pyGSTi to create a report that returns a detailed description of the gate errors. From this report we can extract the process matrices, the contribution of each type of error in the gates and the average gate fidelities.

The error bars for the average gate fidelities are expected to scale as $O(1/L\sqrt{N_{\mathrm{reps}}})$, where L is the depth of the circuits (maximum number of gates on each circuit) and N_{reps} is the number of total repetitions for each circuit. For the chosen values of $L = 8$ and $N_{\mathrm{reps}} \approx 100$, the expected uncertainties are $\approx 1\%$. The GST experiments with magnetic drive ran for 16 hours, and 24 hours for electric drive. During that time we did not perform any recalibration protocol (frequency or readout retuning). The model violation remained relatively low in both cases $\sigma_{\mathrm{NMR}} = 10.35$ and $\sigma_{\mathrm{NER}} = 35.94$.

Coherent errors dominate the error rates (Table S2 and Table S3) in both magnetic and electric drive cases. These errors result from over/under rotations of the qubit and can be corrected by adjusting the duration of specific pulses ($\mathbb{I}$, $X_{\pi/2}$, and $Y_{\pi/2}$). Stochastic errors remained minimal, for both NMR and NER drive. This outcome is expected since the maximum gate set length (L = 8) ensured that the longest pulse sequences were approximately 2 ms for NMR and 10 ms for NER. These durations are well below the dephasing times ($T^*_{2n+} \approx 29$ ms).

In the main manuscript, we quoted the commonly used ‘average fidelity’ values, which reflect the metric typically found from e.g. randomised benchmarking experiments. It is important to note that the relation between the errors listed in Table S2 and Table S3 (Hamiltonian and stochastic) and the average fidelity is non-trivial and potentially confusing. H errors correspond to amplitudes, whereas S errors correspond to probabilities [8]. H errors should be squared when used to estimate a fidelity; furthermore, different H errors may interfere constructively or destructively. Therefore, even when H errors appear to be the largest, this does not immediately imply that the average fidelity can be substantially improved by recalibrating the gates.

Gate	H_X(%)	H_Y(%)	H_Z(%)	S_X(%)	S_Y(%)	S_Z(%)	$\bar{\epsilon}$(%)	ϵ_{tot}(%)
I	0.87(64)	1.1(6)	8.0(6)	0.001(73)	0.14(72)	1.7(6)	1.65(44)	10(2)
$X_{\pi/2}$	-0.92(54)	0.68(48)	0.68(48)	0.02(3)	0.21(23)	0.09(23)	0.24(26)	1.7(1)
$Y_{\pi/2}$	0.67(47)	-0.63(43)	-0.67(48)	0.02(3)	0.0001(32)	0.01(25)	0.04(27)	1.2(1)

TABLE S2: Error rates for the elementary Hamiltonian (H) and stochastic (S) error generators, used to calculate the average gate infidelity $\bar{\epsilon}$ and total error ϵ_{tot} for NER 1Q-GST. Here, $\bar{\epsilon}(\%) = 100 - F(\%)$ where $F(\%)$ is the average gate fidelity reported in Tab.II of the main text.

Gate	H_X(%)	H_Y(%)	H_Z(%)	S_X(%)	S_Y(%)	S_Z(%)	$\bar{\epsilon}$(%)	ϵ_{tot}(%)
I	0.36(51)	0.16(51)	7.1(4)	0.01(50)	0.34(49)	0.0001(30)	0.58(30)	7(1)
$X_{\pi/2}$	0.85(48)	0.47(40)	0.47(40)	0.16(29)	0.0007(23)	0.0003(23)	0.12(25)	1.3(9)
$Y_{\pi/2}$	0.47(40)	0.80(39)	-0.47(42)	0.01(20)	0.12(28)	0.11(20)	0.18(24)	1.3(8)

TABLE S3: Error rates for the elementary Hamiltonian (H) and stochastic (S) error generators, used to calculate the average gate infidelity $\bar{\epsilon}$ and total error ϵ_{tot} for NMR 1Q-GST. Here, $\bar{\epsilon}(\%) = 100 - F(\%)$ where $F(\%)$ is the average gate fidelity reported in Tab.II of the main text.

SUPPLEMENTARY NOTE 12: VOLTAGE AND FREQUENCY FLUCTUATIONS WITH LABORATORY TEMPERATURE

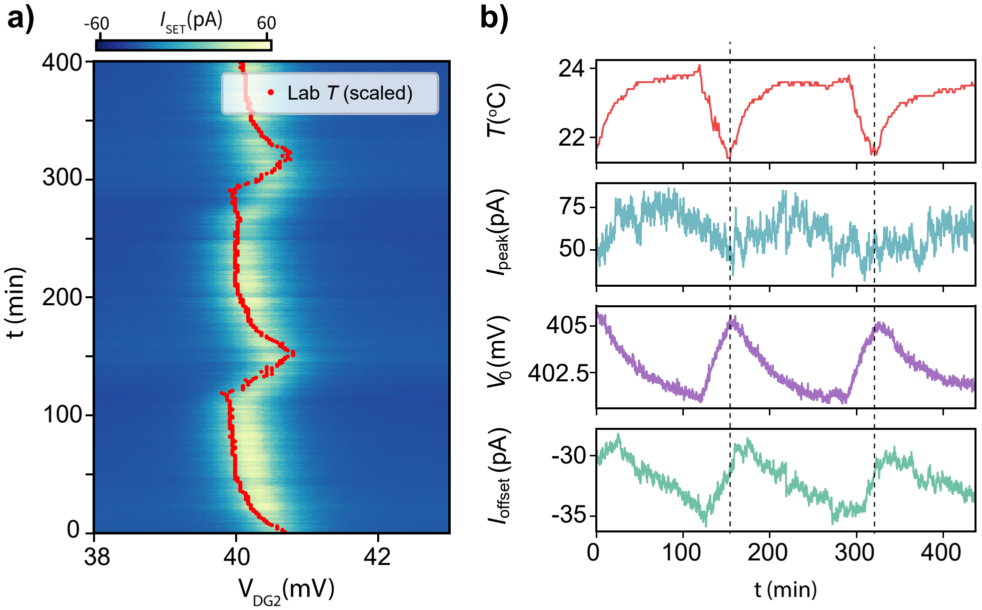

FIG. S13: Temperature-correlated voltage drifts sensed by the SET. a) Line scans across a Coulomb peak (CP) over the course of 6 hours. We sweep the voltage of a donor gate V_{DG2} to track a CP over time and superimpose the lab temperature (red dots). The values of the temperature are rescaled to make the correlation between the temperature and CP drifts, visible to the reader. **b)** Individual SET current traces are fitted using a Gaussian function (explained in main text), and the resulting fitting parameters reveal oscillations in I_{peak}, V_0 and I_{offset} which match the period of oscillations in the temperature T, as highlighted by the black dashed lines.

The temperature in our laboratory fluctuates by up to $2\,^{\circ}\mathrm{C}$ as a consequence of a controller that switches on/off the air conditioning unit with a typical period of two hours. This temperature fluctuation affects several experimental observables, such as the SET current. To investigate this relationship, we tracked the current of the SET by scanning over a Coulomb peak with one of the donor gates (DG2) over the course of $\approx$ 6 hours, while simultaneously measuring the temperature near the measurement equipment, using a EL-GFX-DTP data logger with a Thermistor Probe. Figure S13 a shows the periodic drift of the Coulomb peak over time, following the periodically fluctuating lab temperature, plotted with the red dots. Fitting individual traces with a Gaussian function $I_{\mathrm{peak}} \exp\left(-(V_{\mathrm{DG2}} - V_0)^2/2\sigma^2\right) + I_{\mathrm{offset}}$ reveals that these fluctuations can be resolved in the free parameters of the fit, including the height of the Coulomb peak I_{peak}, the offset current I_{offset}, and the center of the Coulomb peak V_0, as shown in Figure S13 b.

Drifts in the Coulomb peaks and SET current can be explained by changes in voltages applied to the device, which modify the electrochemical potential of the SET island. We investigated whether the temperature affects directly the DC voltage source (SRS SIM928), or the 1:8 resistive voltage dividers used between the source and the device. In both cases, we set the source to output $10\,\mathrm{V}$. Figure S14 a shows the voltage fluctuations directly at the DC source output; Fig. S14 b for the second experiment. For both situations, we plot the deviation for the temperature $\Delta T = T(t) - \overline{T}$ and voltage $\Delta V = V(t) - \overline{V}$ where $\overline{T}$ and $\overline{V}$ are the mean values for the T and V datasets, respectively.

We observe that the oscillations between voltage and temperature are present for both situations, indicating that the fluctuations in the SET from Fig. S13 are likely caused by fluctuations in the voltage supplied to the gates. In the absence of temperature dependence in the resistors of the voltage divider, we would expect a decrease in the oscillations by a factor of 8 compared to the case with no division. Interestingly, we find that the standard deviation $\sigma(1:8) = 58\,\mu\mathrm{V}$ and $\sigma(1:1) = 40\,\mu\mathrm{V}$, which is a measure of the oscillations amplitude, is similar for both. This suggests that the voltage dividers give a significant contribution to the temperature-dependent voltage fluctuations.

Finally, we investigate the impact of these voltage fluctuations on the frequency of the ionized nucleus, since the quadrupolar interaction is sensitive to changes in the electric field applied to the nucleus via the LQSE. We use a

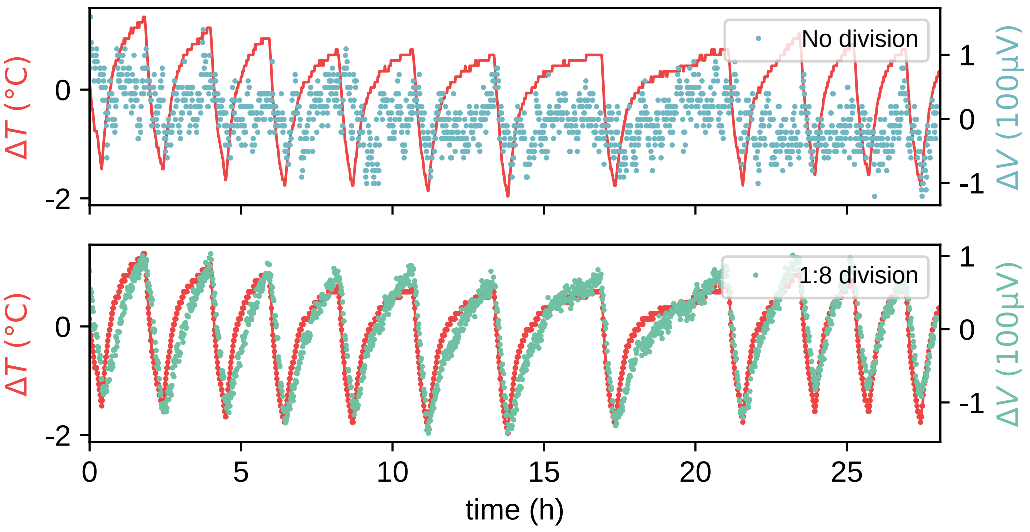

FIG. S14: Correlation between lab temperature and DC voltages. We track the output voltage of a SRS SIM928 voltage source **a)** before and **b)** after a resistive voltage divider (1:8 division) over time, and plot it against the temperature T in the lab measured by a thermistor probe. In both **a)-b)** the voltage source from the SIM module was set to output $10\,\mathrm{V}$. We plot the deviation for the temperature $\Delta T = T - \overline{T}$ and voltage $\Delta V = V(t) - \overline{V}$ where $\overline{T}$ and $\overline{V}$ are the mean values for T and V, respectively.

Ramsey interferometry scheme, where we perform consecutive Ramsey experiments on the ionized nucleus with a fixed free-precession time. The pulse sequence for a Ramsey experiment is given $X_{\pi/2} - \tau - X_{\pi/2}$, where $X_{\pi/2}$ are $\pi/2$ rotations around the x-axis and τ is the time the spin is left free to precess around the xy-plane. When the frequency of the control pulse $f_{\pi/2}$ is detuned from the resonance frequency f_0, the nuclear state probability P oscillates at a frequency $f_{\text{fringe}} = \Delta f = f_{\pi/2} - f_0$, as illustrated in Fig. S15 a. Fixing the value for the free precession time τ, thus allows detecting changes in resonance frequency, as they translate into changes in P, as depicted with the grey shaded area in Fig. S15 a.

We choose $\tau = 12.5\,\mathrm{ms}$ and track the nuclear state probability for $m_I = -5/2$ ($P_{-5/2}$) and the lab temperature T over time, which results in the data presented in Fig. S15 b, showing correlated oscillations for both. This experiments shows that the nuclear spin is capable of detecting temperature fluctuations in the lab, via their effect on the voltage applied to the gates in the device.

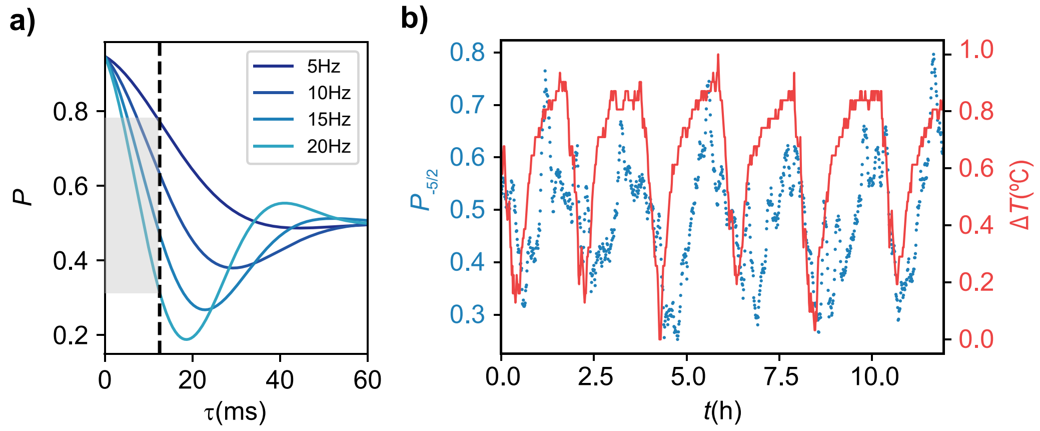

FIG. S15: Temperature-correlated frequency shifts on the ionized ^{123}Sb nucleus. a) Depiction of a Ramsey experiment for different values of detuning Δf (see main text for definition). The decay in the oscillations mimics the decoherence of the spin with a free induction decay time $T^*_{2\mathrm{n}+} = 29\,\mathrm{ms}$ for the $|5/2\rangle \leftrightarrow |7/2\rangle$ in this device. Taking a line cut at a fixed value for the free precession time τ highlights the variation in the nuclear state probability P for the different values of detuning. **b)** We track the nuclear state probability $|-5/2\rangle$ as a function of time for a fixed $\tau = 12.5\,\mathrm{ms}$, and plot it against the normalized lab temperature deviation $\Delta T = T(t) - \overline{T}$, where $\overline{T}$ is the mean value of the temperature dataset. We can use the results from Fig. S14, to correlate the fluctuations from the gate voltages, with changes in the resonance frequency of the ionized donor.

[1] R. Savytskyy, T. Botzem, I. Fernandez de Fuentes, B. Joecker, J. J. Pla, F. E. Hudson, K. M. Itoh, A. M. Jakob, B. C. Johnson, D. N. Jamieson, *et al.*, An electrically driven single-atom "flip-flop" qubit, Science Advances **9**, eadd9408 (2023).

[2] B. Joecker, H. G. Stemp, I. F. de Fuentes, M. A. Johnson, and A. Morello, Error channels in quantum nondemolition measurements on spin systems, arXiv preprint arXiv:2307.14103 10.48550/arXiv.2307.14103 (2023).

[3] A. Morello, J. J. Pla, F. A. Zwanenburg, K. W. Chan, K. Y. Tan, H. Huebl, M. Möttönen, C. D. Nugroho, C. Yang, J. A. Van Donkelaar, *et al.*, Single-shot readout of an electron spin in silicon, Nature **467**, 687 (2010).

[4] V. B. Braginsky and F. Y. Khalili, Quantum nondemolition measurements: the route from toys to tools, Reviews of Modern Physics **68**, 1 (1996).

[5] J. J. Pla, K. Y. Tan, J. P. Dehollain, W. H. Lim, J. J. Morton, F. A. Zwanenburg, D. N. Jamieson, A. S. Dzurak, and A. Morello, High-fidelity readout and control of a nuclear spin qubit in silicon, Nature **496**, 334 (2013).

[6] S. J. Hile, L. Fricke, M. G. House, E. Peretz, C. Y. Chen, Y. Wang, M. Broome, S. K. Gorman, J. G. Keizer, R. Rahman, *et al.*, Addressable electron spin resonance using donors and donor molecules in silicon, Science Advances **4**, eaaq1459 (2018).

[7] D. P. Franke, M. P. Pflüger, P.-A. Mortemousque, K. M. Itoh, and M. S. Brandt, Quadrupolar effects on nuclear spins of neutral arsenic donors in silicon, Physical Review B **93**, 161303 (2016).

[8] M. T. Mądzik, S. Asaad, A. Youssry, B. Joecker, K. M. Rudinger, E. Nielsen, K. C. Young, T. J. Proctor, A. D. Baczewski, A. Laucht, *et al.*, Precision tomography of a three-qubit donor quantum processor in silicon, Nature **601**, 348 (2022).

[9] S. Asaad, V. Mourik, B. Joecker, M. A. Johnson, A. D. Baczewski, H. R. Firgau, M. T. Mądzik, V. Schmitt, J. J. Pla, F. E. Hudson, *et al.*, Coherent electrical control of a single high-spin nucleus in silicon, Nature **579**, 205 (2020).

[10] J. Dehollain, J. Pla, E. Siew, K. Tan, A. Dzurak, and A. Morello, Nanoscale broadband transmission lines for spin qubit control, Nanotechnology **24**, 015202 (2012).

[11] C. Adambukulam, V. Sewani, H. Stemp, S. Asaad, M. Mądzik, A. Morello, and A. Laucht, An ultra-stable 1.5 T permanent magnet assembly for qubit experiments at cryogenic temperatures, Review of Scientific Instruments **92**, 085106 (2021).

[12] R. Kalra, A. Laucht, J. P. Dehollain, D. Bar, S. Freer, S. Simmons, J. T. Muhonen, and A. Morello, Vibration-induced electrical noise in a cryogen-free dilution refrigerator: Characterization, mitigation, and impact on qubit coherence, Review of Scientific Instruments **87**, 073905 (2016).

[13] S. Asaad and M. Johnson, Silq measurement software (2017).

[14] J. H. Nielsen, W. H. Nielsen, M. Astafev, alexcjohnson, D. Vogel, sohail chatoor, G. Ungaretti, MerlinSmiles, Adriaan, S. Pauka, P. Eendebak, qSaevar, P. Eendebak, R. van Gulik, N. Pearson, damazter, A. Corna, S. Droege, damazter2, Thorvald-Larsen, A. Geller, euchas, V. Hartong, S. Asaad, C. Granade, L. Drmić, S. Borghardt, and mltls, Qcodes/qcodes: Qcodes 0.2.1 (2019).

[15] T. Kushida and K. Saiki, Shift of nuclear quadrupole resonance frequency by electric field, Physical Review Letters **7**, 9

(1961).
[16] J. Armstrong, N. Bloembergen, and D. Gill, Linear effect of applied electric field on nuclear quadrupole resonance, Physical Review Letters **7**, 11 (1961).
[17] R. Dixon and N. Bloembergen, Linear Electric Shifts in the Nuclear Quadrupole interaction in al_2o_3, Physical Review **135**, A1669 (1964).
[18] R. Rahman, C. J. Wellard, F. R. Bradbury, M. Prada, J. H. Cole, G. Klimeck, and L. C. Hollenberg, High precision quantum control of single donor spins in silicon, Physical Review Letters **99**, 036403 (2007).
[19] F. R. Bradbury, A. M. Tyryshkin, G. Sabouret, J. Bokor, T. Schenkel, and S. A. Lyon, Stark tuning of donor electron spins in silicon, Physical Review Letters **97**, 176404 (2006).
[20] S. B. Tenberg, S. Asaad, M. T. Mądzik, M. A. Johnson, B. Joecker, A. Laucht, F. E. Hudson, K. M. Itoh, A. M. Jakob, B. C. Johnson, *et al.*, Electron spin relaxation of single phosphorus donors in metal-oxide-semiconductor nanoscale devices, Physical Review B **99**, 205306 (2019).
[21] E. Nielsen, K. Rudinger, T. Proctor, A. Russo, K. Young, and R. Blume-Kohout, Probing quantum processor performance with pyGSTi, Quantum Science and Technology **5**, 044002 (2020).
