# Supplementary material for: Navigating the 16-dimensional Hilbert space of a high-spin donor qudit with electric and magnetic fields
Source: Nat Commun. 2024 Feb 14;15:1380. doi: 10.1038/s41467-024-45368-y (PMC11258329; doi:10.1038/s41467-024-45368-y)
Supplement: Supplementary file 2 — Peer Review File [file 41467_2024_45368_MOESM2_ESM.pdf]

nature portfolio

Peer Review File

Navigating the 16-dimensional Hilbert space of a high-spin donor qudit with electric and magnetic fields

Open Access This file is licensed under a Creative Commons Attribution 4.0 International License, which permits use, sharing, adaptation, distribution and reproduction in any medium or format, as long as you give appropriate credit to the original author(s) and the source, provide a link to the Creative Commons license, and indicate if changes were made. In the cases where the authors are anonymous, such as is the case for the reports of anonymous peer reviewers, author attribution should be to 'Anonymous Referee' followed by a clear attribution to the source work. The images or other third party material in this file are included in the article's Creative Commons license, unless indicated otherwise in a credit line to the material. If material is not included in the article's Creative Commons license and your intended use is not permitted by statutory regulation or exceeds the permitted use, you will need to obtain permission directly from the copyright holder. To view a copy of this license, visit http://creativecommons.org/licenses/by/4.0/.

Editorial Note: This manuscript has been previously reviewed at another journal that is not operating a transparent peer review scheme. This document only contains reviewer comments and rebuttal letters for versions considered at *Nature Communications.*

REVIEWERS' COMMENTS

Reviewer #1 (Remarks to the Author):

I appreciate the changes the authors have made to address the questions and concerns raised by the reviewers. Although key technologies of this work (electric and magnetic control of a combined electron and nuclear spin of a 123Sb donor) and the main results (Figs. 1-3) have been already reported by the same group in Assad et al., Nature 579, 205-209 (2020), coherence and gate fidelity characterization presented in this work demonstrate a potential of the system to be used as a qudits device in the future. Therefore, I recommend publication in Nature Communications.
